# Tankyrase inhibition impairs directional migration and invasion of lung cancer cells by affecting microtubule dynamics and polarity signals

**DOI:** 10.1186/s12915-016-0226-9

**Published:** 2016-01-19

**Authors:** Barbara Lupo, Jorge Vialard, Francesco Sassi, Patrick Angibaud, Alberto Puliafito, Emanuela Pupo, Letizia Lanzetti, Paolo M. Comoglio, Andrea Bertotti, Livio Trusolino

**Affiliations:** Department of Oncology, University of Torino Medical School, 10060 Candiolo, Torino Italy; Laboratory of Translational Cancer Medicine, Candiolo Cancer Institute – FPO IRCCS, Strada Provinciale 142, km 3.95, 10060 Candiolo, Torino Italy; Janssen Research & Development, a Division of Janssen Pharmaceutica NV, 2340 Beerse, Belgium; Janssen Research & Development, a Division of Janssen-Cilag, 27106 Val-de-Reuil, Cedex France; Laboratory of Cell Migration, Candiolo Cancer Institute – FPO IRCCS, 10060 Candiolo, Torino Italy; Laboratory of Membrane Trafficking, Candiolo Cancer Institute – FPO IRCCS, 10060 Candiolo, Torino Italy; Experimental Clinical Molecular Oncology, Candiolo Cancer Institute – FPO IRCCS, 10060 Candiolo, Torino Italy; Istituto Nazionale di Biostrutture e Biosistemi, INBB, 00136 Rome, Italy

**Keywords:** Cancer cell invasion, Cell migration, HGF, Microtubules, Polarity signals, Tankyrase

## Abstract

**Background:**

Tankyrases are poly(adenosine diphosphate)-ribose polymerases that contribute to biological processes as diverse as modulation of Wnt signaling, telomere maintenance, vesicle trafficking, and microtubule-dependent spindle pole assembly during mitosis. At interphase, polarized reshaping of the microtubule network fosters oriented cell migration. This is attained by association of adenomatous polyposis coli with the plus end of microtubules at the cortex of cell membrane protrusions and microtubule-based centrosome reorientation towards the migrating front.

**Results:**

Here we report a new function for tankyrases, namely, regulation of directional cell locomotion. Using a panel of lung cancer cell lines as a model system, we found that abrogation of tankyrase activity by two different, structurally unrelated small-molecule inhibitors (one introduced and characterized here for the first time) or by RNA interference-based genetic silencing weakened cell migration, invasion, and directional movement induced by the motogenic cytokine hepatocyte growth factor. Mechanistically, the anti-invasive outcome of tankyrase inhibition could be ascribed to sequential deterioration of the distinct events that govern cell directional sensing. In particular, tankyrase blockade negatively impacted (1) microtubule dynamic instability; (2) adenomatous polyposis coli plasma membrane targeting; and (3) centrosome reorientation.

**Conclusions:**

Collectively, these findings uncover an unanticipated role for tankyrases in influencing at multiple levels the interphase dynamics of the microtubule network and the subcellular distribution of related polarity signals. These results encourage the further exploration of tankyrase inhibitors as therapeutic tools to oppose dissemination and metastasis of cancer cells.

**Electronic supplementary material:**

The online version of this article (doi:10.1186/s12915-016-0226-9) contains supplementary material, which is available to authorized users.

## Background

In the last decade two enzymes belonging to the poly(adenosine diphosphate)-ribose polymerase (PARP) superfamily, tankyrase 1 (TNKS) and 2 (TNKS2), were identified as key regulators of spindle pole assembly through poly(adenosine diphosphate)-ribosylation (PARsylation) of several microtubule-related proteins within the spindle apparatus [1, 2]. Poly (adenosine diphosphate)-ribose (PAR) units have also been accredited as integral spindle constituents, with TNKS and TNKS2 (TNKS/2 hereinafter) being the prime regulators of spindle-associated PAR synthesis [3]. TNKS/2 downregulation is consistently reported to yield aberrant mitotic phenotypes, including microtubule defects and supernumerary spindles [4]. TNKS/2 are also required for proper sister telomere resolution [5] and centrosome function [6, 7]. Altogether, these observations added to the archetypal function of these enzymes as positive regulators of telomere homeostasis [8, 9] and spurred a growing interest in neutralizing their activity to induce spindle dysfunction and disable the mitotic engine in cancer cells [10, 11]. Independent studies have also shown that TNKS/2 positively regulate the Wnt/β-catenin signaling pathway. In particular, TNKS/2 have been reported to inhibit the β-catenin destruction complex by promoting the degradation of its rate-limiting component, axin1 [12]. Consequently, β-catenin remains unbridled and is allowed to enter the nucleus, where its gene program is released [12].

The multifaceted activities exerted by tankyrases can be explained by the vast number and heterogeneity of putative TNKS/2 substrates: in silico analyses have put forward hundreds of candidates [13], some of which—including mitotic regulators, transcription factors, and signaling adaptors—have been validated as true TNKS/2 binders by classic protein–protein interaction assays [13–17]. However, the biological relevance of most such interactions still require experimental scrutiny, suggesting that other, as yet unappreciated, functions of TNKS/2 will emerge soon.

In this work, we provide evidence of a novel role for TNKS/2 in regulating directional migration. By using two distinct and structurally unrelated inhibitors, including a new investigational compound for which we provide original structural, pharmacological, and biochemical characterization, we found that abrogation of TNKS/2 activity markedly weakened cancer cell motility owing to perturbation of recognized microtubule-dependent routes that govern cell-oriented locomotion. The finding that TNKS/2 blockade impacts microtubule-based cellular processes not only at mitosis but also in interphase cells expands our knowledge about TNKS/2 functions at the cellular level and should hasten the preclinical development of TNKS/2 inhibitors for applicative purposes.

## Results

### Structural, pharmacological, and biochemical characterization of JNJ-BJ, a novel TNKS/2 inhibitor

**Fig. 1:**
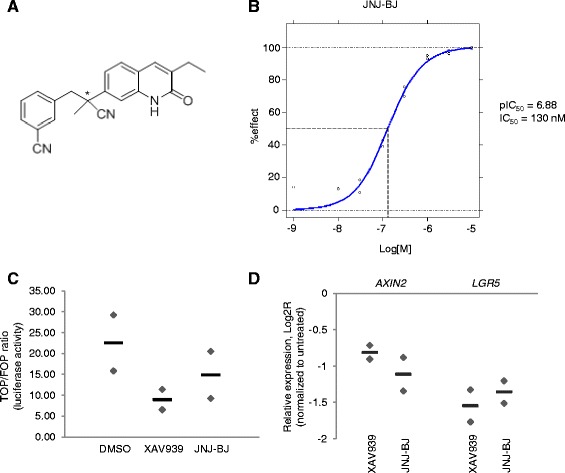
Structural, pharmacological, and biochemical characterization of JNJ-BJ. **a** Chemical structure of JNJ-BJ. The compound is the first eluted enantiomer of a 3-ethylquinolinone bearing a chiral center linked to the C-7 carbon atom ((R or S)-3-[2-cyano-2-(3-ethyl-2-oxo-1H-quinolin-7-yl)propyl]benzonitrile). **b** Tankyrase (TNKS) autoribosylation assay. A recombinant His-tagged human TNKS2 poly(adenosine diphosphate)-ribose polymerase domain was produced in a baculovirus/insect cell expression system. The purified protein was bound to a 384-well Ni^2+^-coated Flashplate and incubated for 120 min with NADH3/NAD in the presence of increasing concentration of JNJ-BJ. The radioactive signal was measured using a scintillation reader. **c** Assessment of β-catenin/TCF transcriptional activity through a TOPflash reporter assay in adenomatous polyposis coli-mutant DLD1 colorectal cancer cells. Cells were treated with TNKS/2 inhibitors (10 μM) for 24 h. Results are expressed as TOP/FOP ratio and represent the average (*lines*) ± range of two independent experiments (*diamonds*), each performed in technical quadruplicate. **d** Expression of *AXIN2* and *LGR5* transcripts in DLD1 cells treated with TNKS/2 inhibitors (10 μM) for 24 h. Results are the average (*lines*) of two independent experiments (*diamonds*), each performed in technical triplicate. Raw data for panels c and d are shown in Additional file 2. *DMSO* dimethyl sulfoxide, *IC*_*50*_ half-maximal inhibitory concentrations

XAV939 is a pyrimidine derivative that inhibits TNKS/2 by binding to the nicotinamide pocket of the enzymes, with half-maximal inhibitory concentrations (IC50) of 0.011 μM and 0.004 μM, respectively [12, 18]. JNJ-BJ is the first eluted enantiomer of a 3-ethylquinolinone (1A) and, like XAV939, competes with nicotinamide binding to tankyrases. When tested in an auto-PARsylation assay against the recombinant, baculovirus-expressed PARP domain of TNKS2, JNJ-BJ displayed an IC50 of 0.13 μM (pIC50 6.88; Fig. 1b). Details on the compound synthesis scheme are provided in Additional file 1.

A characteristic readout of TNKS/2 inhibition is a reduction in β-catenin-dependent signaling in cells with a hyperactive Wnt pathway [12]. Coherent with the inhibitory activity towards purified TNKS2, treatment of adenomatous polyposis coli (APC)-mutant DLD1 colorectal cancer cells with JNJ-BJ impaired Wnt-driven transcriptional responses, as assessed by both a TOPflash luciferase reporter assay (Fig. 1c; raw data in Additional file 2) and reverse transcription quantitative polymerase chain reaction (RT-qPCR) analysis of the expression of established β-catenin target genes (Fig. 1d; raw data in Additional file 2). As expected, and in accordance with previous findings [12], similar results were obtained with XAV939 (Fig. 1c, d; raw data in Additional file 2).

### TNKS/2 inhibition hampers lung cancer cell invasion and migration in response to hepatocyte growth factor

Although mutations of APC or β-catenin are infrequent in lung cancer, hyperactivation of the Wnt pathway, as evidenced by transcriptional overexpression of Wnt-responsive genes, has been documented in samples from aggressive lung adenocarcinomas [19]. Because TNKS/2 are accredited upstream regulators of the Wnt pathway [12], we initially pursued the idea that interception of TNKS/2 activity might prevent Wnt-induced lung cancer cell dissemination. As a first step, we explored the consequences of TNKS/2 blockade on cell motility in four lung adenocarcinoma cell lines—H322, HCC827, H460, and A549—using XAV939 and JNJ-BJ as tool compounds.

**Fig. 2:**
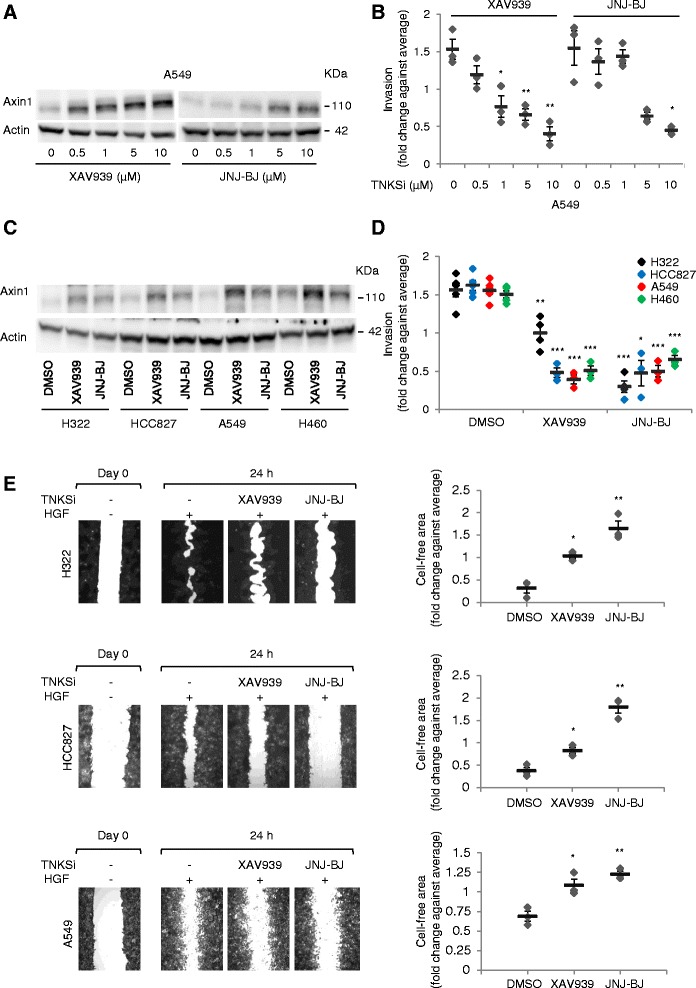
Tankyrase 1 and 2 (TNKS/2) inhibition by XAV939 or JNJ-BJ impairs hepatocyte growth factor (*HGF*)-dependent cell migration and invasion in non-small cell lug cancer (NSCLC) cells. **a** Western blot showing the amount of axin1 in total protein extracts from A549 cells treated with a range of concentrations of XAV939 or JNJ-BJ for 24 h. Actin was used as a loading control. Molecular weights are indicated. **b** Dose-dependent inhibition of HGF-induced invasion in A549 cells by treatment with increasing concentrations of XAV939 or JNJ-BJ (HGF, 50 ng/mL). Quantification was carried out by ImageJ software. *TNKSi* TNKS/2 inhibitor. Data are the means (*lines*) ± standard error of the mean (SEM) of three independent experiments (*diamonds*), each performed in technical duplicate. **c** Western blot analysis showing the levels of axin1 in whole-cell extracts from the indicated NSCLC cell lines. Before protein extraction, cells were incubated with 10 μM XAV939 or JNJ-BJ for 24 h. Actin was used as a loading control. Molecular weights are indicated. **d** ImageJ quantification of HGF-induced invasion in the indicated cell lines upon TNKS/2 blockade (HGF, 50 ng/mL; TNKS/2 inhibitors, 10 μM). Data are the means (*lines*) ± SEM of three or four independent experiments (*diamonds*), each performed in technical duplicate. **e** Representative images of a scratch assay performed with the indicated cell lines in the following experimental conditions (from left to right): non-stimulated control wound (Day 0); HGF-treated cells 24 h after wounding (HGF, 50 ng/mL); HGF-treated and XAV939/JNJ-BJ-treated cells 24 h after wounding (HGF, 50 ng/mL; TNKS/2 inhibitors, 10 μM). Plots on the right depict ImageJ quantification of wound repair inhibition 24 h after scratch. Data are the means (*lines*) ± SEM of three independent experiments (*diamonds*), each performed in technical duplicate or triplicate. *P < 0.05; **P < 0.01; ***P < 0.001 by two-tailed Student’s *t*-test (TNKSi-treated versus untreated [dimethyl sulfoxide, *DMSO*]). Raw data for panels b, d, and e are shown in Additional file 3

To provide proof of concept that TNKS/2 blockade was proficient in lung cancer, A549 cells were treated with increasing concentrations of XAV939 or JNJ-BJ for 24 h and assessed for expression of axin1, which is typically stabilized by TNKS/2 inhibition owing to impaired TNKS/2-mediated PARsylation and consequent protein degradation [12]. Western blot analysis of total cell extracts revealed that both compounds were able to induce a dose-dependent increase of axin1 protein content (Fig. 2a), indicating successful TNKS/2 inactivation. Remarkably, when challenged in Matrigel-coated Transwell systems using hepatocyte growth factor (HGF) as a chemoattractant [20], A549 cells exhibited a dose-dependent reduction in invasive ability following TNKS/2 inactivation by XAV939 or JNJ-BJ (Fig. 2b; raw data in Additional file 3).

Analyses were subsequently extended to the remaining lung cancer cell lines by applying the dose that yielded maximal invasion impairment in the setup experiments (10 μM). In the case of XAV939, 5–10 μM is the standard inhibitor concentration commonly used in biological studies [21, 22]. Consistent with that observed in A549, axin1 was invariably stabilized upon treatment with either compound (Fig. 2c). Similarly, TNKS/2 inactivation compromised HGF-induced chemotactic response to a comparable extent in all the cell lines tested, apart from a weaker activity of XAV939 in H322 (Fig. 2d; raw data in Additional file 3). A decrease in cell invasion was paralleled by reduced migration in wound healing (scratch) assays. With the exception of H460 cells (which proved unsuitable for production of a compact monolayer and were therefore excluded), abrogation of TNKS/2 activity markedly dampened HGF-induced wound closure competence (Fig. 2e; raw data in Additional file 3).

It is worth noting that in these cells we did not observe noticeable anti-proliferative effects following TNKS/2 inhibition, even after a 72 h exposure to drugs (Additional file 4: Figure S1; raw data in Additional file 5). This result is at odds with the established mitotic function of TNKS/2, but is congruent with previous observations showing that TNKS/2 pharmacological inhibition is much less detrimental to cell proliferation than RNA interference (RNAi)-based silencing [4, 5, 12, 21]. Whatever the explanation for this discrepancy, which remains a matter of debate [2, 12, 21], these findings suggest that mechanisms other than a mere growth disadvantage are implicated in the reduced cell motility observed in response to TNKS/2 blockade.

We also employed RNAi as an alternative means of inactivating TNKS/2. In agreement with pharmacologic experiments, productive co-depletion of TNKS and TNKS2 in A549 cells (Additional file 6: Figure S2A and S2B; raw data in Additional file 7) resulted in axin1 stabilization (Additional file 6: Figure S2B) and reduced cell invasion (Additional file 6: Figure S2C; raw data in Additional file 7). Likewise, wound closure ability was lessened by RNAi-mediated TNKS/2 silencing in A549 cells (Additional file 6: Figure S2D; raw data in Additional file 7). Although genetic knockdown of TNKS/2 has been shown to affect cell proliferation, the time frame of Transwell and scratch assays (24 h) was likely sufficiently short not to bias the anti-invasive outcome of TNKS/2 abrogation. In summary, impaired cell invasion proved to be a direct function of increasing compound concentrations and was achieved by two structurally different inhibitors; moreover, TNKS/2 genetic silencing recapitulated the biochemical and biological effects of pharmacologic inhibition. These findings indicate that the impaired chemotactic response is a specific consequence of TNKS/2 disruption.

### The anti-invasive outcome of TNKS/2 inhibition is independent of the Wnt pathway

The anti-invasive and anti-migratory effects produced by TNKS/2 neutralization were consistent with the working hypothesis that blockade of TNKS/2 activity would blunt Wnt-mediated pro-invasive cues. We therefore analyzed whether this weakened chemotactic response was in fact ascribable to interception of Wnt signaling.

First, the TOPflash reporter system was employed to gauge Wnt-dependent transcriptional responses after cell exposure to TNKS/2 inhibitors (for this purpose, we used H322 cells owing to their high amenability to transfection procedures). We found that TNKS/2 inhibition did not affect Wnt transcriptional activity, either basally or upon addition of the canonical Wnt ligand Wnt3a (Additional file 8: Figure S3A; raw data in Additional file 9). As a complementary approach, expression of established Wnt target genes was assessed by RT-qPCR in the whole panel of lung adenocarcinoma cell lines tested in the cell invasion experiments. As shown in Additional file 8: Figure S3B (raw data in Additional file 9), stimulation with Wnt3a led to increased expression of at least some of the target genes, with variable levels of induction in the various cell lines (likely due to cell type-specific differences). Also in this experimental setting, and consistent with the TOPflash assay, cell line-dependent transcription of Wnt3a target genes was not detectably influenced by treatment with TNKS/2 inhibitors (Additional file 8: Figure S3B; raw data in Additional file 9). Finally, Transwell assays demonstrated that cell invasion was not evidently fostered by Wnt3a (Additional file 8: Figure S3C; raw data in Additional file 9), further supporting the irrelevance of Wnt signaling to TNKS/2-related migratory phenotypes in our cellular models.

All in all, Wnt-dependent activities did not substantially enhance cell motility in lung cancer cell lines, nor were they clearly impacted by TNKS/2 inactivation. This implies that the observed effects on migration and invasion likely rely on alternative mechanisms.

### TNKS/2 inhibition impacts the dynamics of formation of cell membrane protrusions

**Fig. 3:**
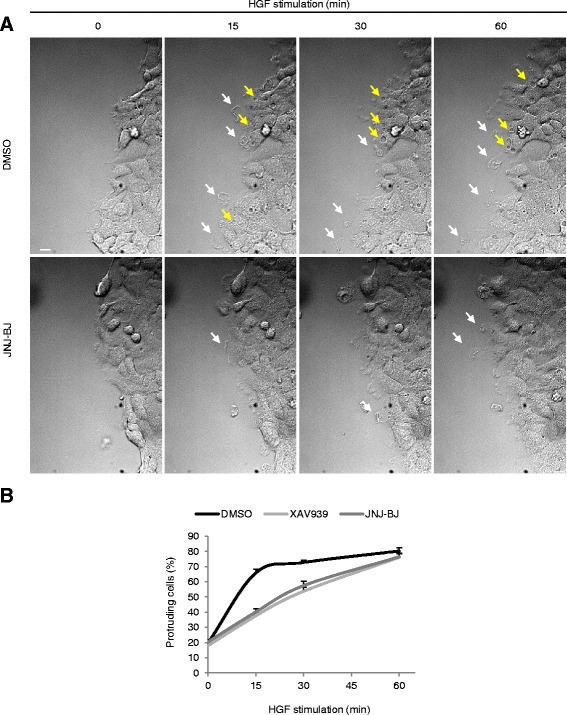
Inactivation of tankyrase 1 and 2 (TNKS/2) weakens protrusive activity and lamellipodia formation. **a** Phase-contrast snapshots extracted from a 1 h time-lapse movie of wounded A549 monolayers treated with hepatocyte growth factor (*HGF*; 50 ng/mL) in the presence or absence of JNJ-BJ (10 μM). *White arrows* indicate membrane projections; *yellow arrows* label circular dorsal ruffles. See Additional file 10: Movie M1 for complete visualization. Scale bar, 7 μm. **b** Quantitation of membrane protrusions in HGF-stimulated wound-edge A549 cells with or without TNKS/2 inhibitors (see “Methods” for details; HGF, 50 ng/mL; XAV939 and JNJ-BJ, 10 μM). Results are expressed as the percentage of protrusion-positive cells ± standard error of the mean. A minimum of 163 cells was counted for each experimental point in three independent experiments (*P* < 0.001 by two-way analysis of variance). Raw data for panel b are shown in Additional file 11. *DMSO* dimethyl sulfoxide

To get better insight into how cell motogenic responses were impacted by TNKS/2 abrogation, wounded A549 monolayers were treated with HGF and monitored for 1 h using time-lapse videomicroscopy in the presence or absence of JNJ-BJ, which was selected as the best performing compound in preliminary experiments (see Fig. 2e). Single images were captured every 12 s to allow for distinct visualization of the migration process (Additional file 10: Movie M1). Figure 3a shows representative time-lapse snapshots, captured 15, 30, and 60 min after HGF stimulation. We noticed that the dynamics of protrusion formation at the wound edge were discernibly slowed by JNJ-BJ. In particular, control cells displayed pronounced membrane ruffling, characterized by repetitive and vigorous bursts of incipient projections at the leading edge (Fig. 3a, white arrows); by contrast, protrusive activity appeared flimsier and membrane extensions subsided quickly in cells treated with JNJ-BJ. Besides the stronger propulsive flows, control cells also developed numerous circular dorsal ruffles (Fig. 3a, yellow arrows), whose function generally implies transition from a static to a motile phenotype [23]; conversely, the formation of such structures was almost completely prevented by JNJ-BJ (Fig. 3a and Additional file 10: Movie M1).

On the basis of such observations, we assumed that TNKS/2 inhibition impaired cell movement by negatively impacting migration dynamics at the leading edge. To complement the time-lapse qualitative information, we quantified membrane extensions in HGF-stimulated A549 cells with or without TNKS/2 inhibitors. As shown in Fig. 3b (raw data in Additional file 11), the proportion of protruding cells was significantly decreased by either compound after 15 and 30 min of HGF exposure. Remarkably, the curves related to TNKS/2-inhibited cells tended to re-align with the curve of control cells after 1 h, suggesting that TNKS/2 blockade hindered early rather than late events of cell migration.

### TNKS/2 inhibition enhances microtubule stability in interphase cells

TNKS/2 couple with the mitotic microtubule circuitry to affect spindle structure and function [4]. As specified earlier, this is accomplished through interaction with various microtubule-related proteins as well as with other spindle-associated targets [1, 2, 4, 15]. We reasoned that analogous functional connections might be extended to interphase microtubule-dependent activities, whose dynamics are intimately related to polarized cell migration [24, 25].

**Fig. 4:**
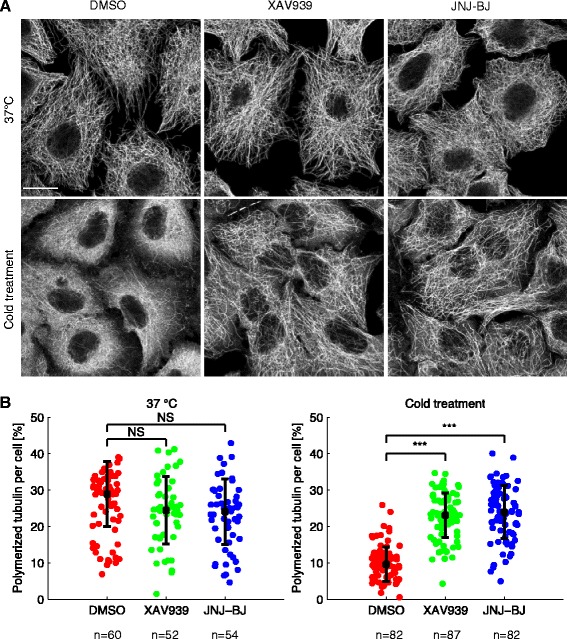
The microtubule network is stabilized by tankyrase 1 and 2 (TNKS/2) pharmacological neutralization. **a** Confocal images and **b** morphometric quantitation of the microtubule network at standard temperature (37 °C) or after cold (4 °C) exposure. Starved A549 cells were pre-incubated for 24 h with or without TNKS/2 inhibitors (10 μM) and then placed on ice for 6 min (cold treatment) before immediate fixation and staining with anti-α-tubulin antibody. Scale bar, 7 μm. Images are representative of one experiment out of two performed. A minimum of 52 cells from at least two different fields were analyzed for content of polymerized tubulin. ***P < 0.001 by two-tailed Student’s *t*-test. *NS* not significant. Raw data for panel b are shown in Additional file 13. *DMSO* dimethyl sulfoxide

Inception of oriented cell movement entails microtubule-dependent reorganization of the cellular architecture to establish a rear–front axis. This asymmetric pattern is supported by the inherent instability of microtubules, which constantly undergo rounds of shrinkage and regrowth [26]. To investigate whether TNKS/2 neutralization interfered with microtubule dynamic instability, we deconstructed the microtubule network in A549 cells by cold treatment (4 °C, 6 min) or nocodazole (1 μM, 5 min). Under basal conditions, microtubules were intact and their organization was similar in both untreated and TNKS/2-inhibited cells (Fig. 4a, b and Additional file 12: Figure S4; raw data for Fig. 4b in Additional file 13). Notably, cold-induced or nocodazole-induced microtubule disassembly was widespread in control cells whereas it was markedly prevented in cells treated with XAV939 or JNJ-BJ (Fig. 4a, b and Additional file 12: Figure S4; raw data for Fig. 4b in Additional file 13). This indicates that microtubules were rendered more stable by TNKS/2 inactivation. RNAi-mediated TNKS/2 depletion recapitulated the phenotype produced by TNKS/2 pharmacologic inactivation (Additional file 14: Figure S5).

The finding that TNKS/2 blockade increased the proportion of stable microtubules suggests that TNKS/2 inhibition might obstruct microtubule-dependent activities implicated in cell polarity and directional migration.

### TNKS/2 inhibition affects centrosome reorientation in migrating cells

Microtubule-related activities are central to polarized cell migration through mechanisms that involve protein targeting to cortical sites and the generation of pulling forces that help reorganize cell architecture in response to chemotactic cues [24, 27, 28]. One hallmark of cell polarization is the relative orientation of the nuclear-centrosome axis with respect to the rear–front axis (which defines the direction of cell migration); in general, this alignment is thought to correlate with the onset of cell migration and to contribute to the establishment of cell polarity by facilitating membrane trafficking from both the Golgi and the endocytic recycling compartments towards the leading edge [29]. Centrosome positioning is largely influenced by microtubule dynamics; regardless of context-dependent idiosyncratic differences, it is apparent that during productive cell locomotion the centrosome relocates in front of the nucleus facing the direction of cell migration [29, 30].

**Fig. 5:**
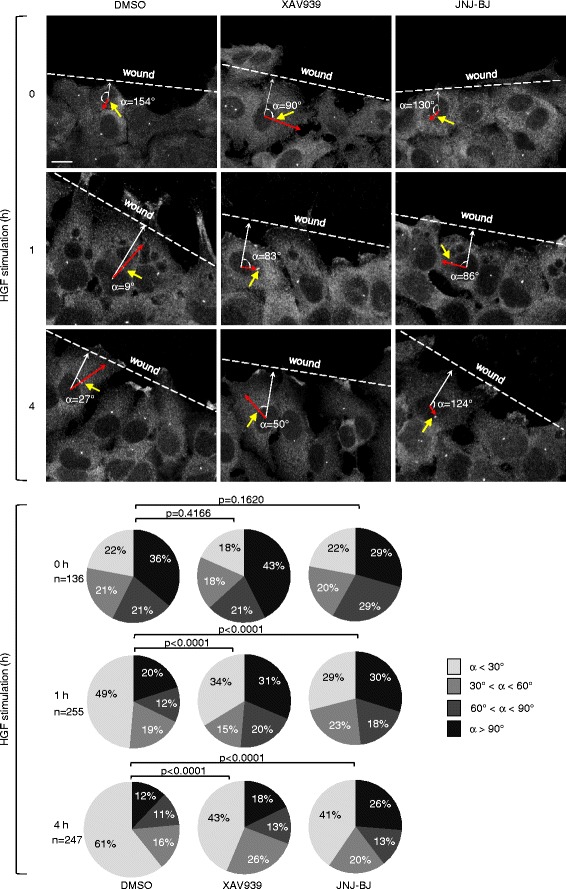
Centrosome reorientation is retarded by tankyrase I and 2 (TNKS/2) inhibitors in migrating cells. Representative confocal images of centrosome localization in migrating A549 cells. Wound-edge A549 cells, with or without TNKS/2 inhibitors (10 μM), were stimulated with hepatocyte growth factor (*HGF*) (50 ng/mL) for 4 h. Immunofluorescence staining of centrosomes was carried out using anti-γ-tubulin antibody. Centrosome polarization was assessed at the indicated time points. Pie charts display the percentage distribution of distinct angles (*α*) formed by the nuclear-centrosome axis (*red arrows*) and a line perpendicular to the wound (*white arrows*). Centrosomes were considered oriented when α < 30°. A minimum of 136 cells was analyzed for centrosome polarization in three independent experiments. *P*-values were calculated by the Chi-square test using Graphpad Quickcalcs software. Scale bar, 7 μm. Raw data are shown in Additional file 15. *DMSO* dimethyl sulfoxide

On such premises, we explored whether TNKS/2 inhibition could perturb microtubule-dependent establishment of cell asymmetry by measuring the amount of reoriented centrosomes in wound-edge A549 cells following induction of migration by HGF. Based on previously published studies [31], centrosomes were scored as “fully polarized” if the angle between the nuclear-centrosome axis (Fig. 5, red arrows) and the front-back axis (Fig. 5, white arrows) was less than 30°, which vouches for centrosome dwelling within the forward-facing quadrant (raw data in Additional file 15). In HGF-treated cells without TNKS/2 inhibitors, centrosome location shifted from an essentially random distribution around the nucleus to a biased rearrangement along the migration axis, with polarization angles reflecting a progressive degree of cell orientation over time (Fig. 5). The increase of fully polarized centrosomes was accompanied by a concomitant reduction in the number of untailored centrosomes displaying angles greater than 60° (Fig. 5). Remarkably, HGF-induced acquisition of the polarized phenotype was antagonized by treatment with either TNKS/2 inhibitor. At 1 h after cell wounding, almost 50 % of control cells were fully polarized, compared to only 34 % and 29 % of XAV939-treated and JNJ-BJ-treated cells, respectively; also the decline of non-polarized centrosomes was not as manifest as that observed in control cells (Fig. 5). After 4 h, 60 % of control cells showed fully oriented centrosomes, as opposed to nearly 40 % of TNKS/2-inhibited cells (Fig. 5). Akin to TNKS/2 pharmacologic blockade, genetic knockdown of both TNKS and TNKS2 in A549 cells disturbed centrosome repositioning upon HGF stimulus (Additional file 16: Figure S6). Collectively, these results indicate that TNKS/2 inactivation results in perturbed centrosome reorientation in migrating cells, with delayed alignment along the rear–front axis.

### TNKS/2 inhibition retards APC recruitment at the leading edge

**Fig. 6:**
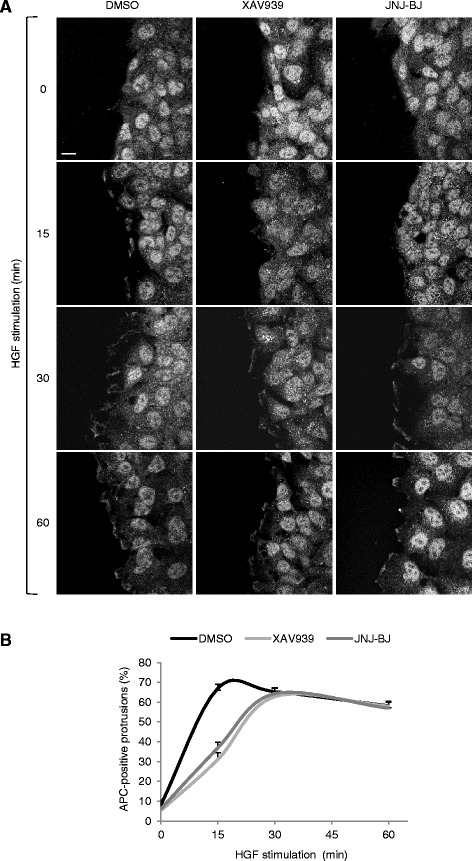
Tankyrase 1 and 2 (TNKS/2) inhibitors delay the formation of cortical adenomatous polyposis coli (APC) clusters in migrating cells. **a** Confocal images of serum-starved, wound-edge A549 cells, either untreated or treated with TNKS/2 inhibitors (10 μM) and then subject to a time course of hepatocyte growth factor (*HGF*) stimulation (HGF, 50 mg/mL). Cells were fixed and stained with anti-APC antibody. **b** Quantitation of APC-decorated protrusions. The curves represent APC enrichment in cortical areas over time. Results are expressed as the percentage of APC-positive protrusion ± standard error of the mean. A minimum of 163 cells was counted for each experimental condition in three independent experiments (*P* < 0.001 by two-way analysis of variance). Scale bar, 7 μm. Raw data for panel b are shown in Additional file 17. *DMSO* dimethyl sulfoxide

Adenomatous polyposis coli (APC) is a strategic cytoskeletal coordinator of migration directionality owing to its recruitment to microtubule-dependent clusters at protrusive areas. This is thought to regulate microtubule anchoring at the cell cortex and consequent centrosome rearrangement [32, 33]. With this in mind, we assessed HGF-induced redistribution of APC in wounded A549 cell monolayers, either left untreated or exposed to TNKS/2 inhibitors. Immunofluorescence staining showed a diffuse APC cytoplasmic distribution along with an intense nuclear signal (Fig. 6a), consistent with the notion that APC shuttles between the nucleus and the cytoplasm to assist β-catenin nuclear export [34]. Immediately after wounding, APC was evenly distributed throughout the cell monolayer and in cells closely adjacent to the wound area (Fig. 6a). APC redistribution in cortical clusters at the wound edge was evident in control cells as early as 15 min after HGF stimulation and persisted up to 1 h (Fig. 6a). By contrast, treatment with TNKS/2 inhibitors led to a marked attenuation of APC membrane targeting at the 15 min time point after HGF stimulation. Morphometric quantitation revealed that the number of APC-positive protrusions was reduced by TNKS/2 inactivation also when normalized against the number of total protrusions (Fig. 6b; raw data in Additional file 17). This indicates that the impairment of APC membrane relocalization was not a mere consequence of lessened membrane ruffling secondary to TNKS/2 blockade. Of note, the representative curves of control and TNKS/2-inhibited cells tended to readjust over time: in fact, APC-decorated lamellipodia were equally represented at 30 min and 1 h post-wounding in all the conditions tested (Fig. 6a, b). Thus, TNKS/2 inhibition deferred APC membrane redistribution in response to HGF, which integrates with our previous data about retarded centrosome repositioning. The fact that APC localization in protrusions was only transiently retarded, whereas centrosome reorientation was impaired for longer times, is congruent with the notion that, once cell polarization is established, positive feedback loops initiate between the actin-rich cortex and the microtubule cytoskeleton to maintain and reinforce the existing polarity axis [35]. Therefore, while APC relocation is required for centrosome rearrangement, the persistence of such a tailored phenotype is likely sustained by processes that engage proteins and network interactions other than APC, together with the microtubule cytoskeleton. Remarkably, impaired accumulation of APC at the protrusive front was even exacerbated by RNAi-mediated TNKS/2 knockdown; indeed, HGF-induced recruitment of APC at cortical areas was prevented at all the time points in A549 wound-edge cells transduced with TNKS- and TNKS2- short hairpin RNAs (Additional file 18: Figure S7).

Finally, to get further insight into how TNKS/2 may regulate APC-dependent microtubule dynamics, we analyzed the subcellular distribution of TNKS and APC in migrating A549 cells. We found that both APC and TNKS were recruited at the leading edge upon HGF stimulation (Additional file 19: Figure S8). Similar to APC, TNKS enrichment at membrane protrusions was impaired in the presence of TNKS/2 inhibitors (Additional file 19: Figure S8). TNKS staining was specific, because TNKS/2 blockade also induced the formation of TNKS-enriched puncta, a reported phenotype of TNKS/2-inhibited cells [36] (Additional file 19: Figure S8).

The fact that TNKS follows subcellular dynamics similar to those experienced by APC and the experimental observation that persistent obliteration of TNKS/2 by genetic silencing durably precludes APC relocation at the leading edge reinforce the hypothesis that TNKS/2 are implicated in the establishment of cell polarization during oriented locomotion. Taken together, these observations allow us to draw a coherent scenario whereby TNKS/2 blockade appears to interfere sequentially with regulated events that, in space and time, orchestrate the establishment of cellular polarity. This perturbation of cell polarity is facilitated by the enhanced microtubule stability produced by TNKS/2 inactivation.

## Discussion

The PARPs TNKS and TNKS2 were initially identified as key players in telomere homeostasis through inhibition of TRF1, a negative regulator of telomere length [8, 9]. Independent lines of evidence also accredited TNKS/2 as positive regulators of microtubule-dependent mitotic events through their interaction with a number of spindle-associated proteins (which assures proper bipolar spindle formation) [1, 2] as well as through PARsylation of key centrosomal targets (which regulates accurate microtubule-aster formation) [6, 7]. Finally, TNKS/2 have recently been identified as upstream components of the Wnt/β-catenin pathway, which encourages cell proliferation by intensification of β-catenin transcriptional activities [12]. The notion that tankyrases have versatile activities in processes that, when gone awry, invariably lead to aberrant cell growth, has spurred the development of several TNKS/2 inhibitors [11, 37, 38].

Here we report a new function of TNKS/2 that does not involve regulation of cell cycle entry or cell division, but rather influences cell polarity and directional cell locomotion. Indeed, we found that pharmacological inhibition of TNKS/2 dampens microtubule-dependent cell chemotactic responses. Because recent data have linked Wnt transcriptional signatures to the metastatic competence of lung adenocarcinoma cells [19], our initial working hypothesis was that the anti-migratory outcome of TNKS/2 inhibition might be ascribed to impaired Wnt signaling. However, in our experimental setup, TNKS/2 inhibition did not substantially affect Wnt-associated responses and Wnt stimulation did not detectably promote cell invasion, indicating that the decline of cell motility as a consequence of TNKS/2 inhibition occurred irrespective of Wnt activity. This hints that TNKS/2-directed drugs are likely to prove effective as inhibitors of Wnt signals only in those tumors that display constitutive Wnt activation on a genetic basis, such as colorectal cancer. Indeed, there is cumulative evidence that pharmacologic neutralization of TNKS/2 activity proficiently (albeit not uniformly) impairs the growth of APC-mutant colorectal cancer cells by attenuating Wnt-mediated signals [12, 39, 40].

None of the established functions of TNKS/2 can explain the less motile phenotype observed in TNKS/2-inhibited cells. Live imaging of migrating cells allowed us to detect less intense and less persistent protrusive activities upon TNKS/2 blockade, likely reflecting different cytoskeletal dynamics at the leading edge. On the one hand, TNKS/2 play a documented role in the regulation of microtubules during mitosis. On the other hand, many facets of cell protrusions—including orientation and persistence—are determined by microtubule-related activities, and disruption of the microtubule network impairs protruding activity in several cellular contexts [24, 31, 41–43]. We therefore sought to extend the connection between TNKS/2 and the microtubule network from mitotic to interphase-related processes, with a specific focus on whether and how TNKS/2 may influence microtubule-dependent cell polarization as a prelude to directional movement.

The regulation of microtubule dynamics during cell polarization is complex. In general, it is believed that a wide range of polarity cues, including intracellular signals such as Cdc42 and soluble morphogens such as motogenic growth factors, converge to activate transducers that phosphorylate and inhibit glycogen synthase kinase 3 beta (GSK3β) [24, 44]. This leads to APC interaction with the microtubule plus ends at the leading edge, which stabilizes the microtubule network specifically at the protrusive areas and generates pulling forces that reorient the centrosome and align it along the rear–front axis [32]. Successful implementation of all these events requires consecutive episodes of microtubule catastrophes (depolymerization) and rescues (repolymerization) in order to grant microtubules with local “search-and-capture” activity. Accordingly, it has been demonstrated that several proteins that stall microtubule dynamics exert anti-invasive effects by preventing microtubules from probing outward, resulting in faulty capture to cortical sites and reduced receptiveness of chemotactic inputs [45, 46]. We found that TNKS/2 blockade impacted some of these aspects of cell directional sensing. First, cells treated with TNKS/2 inhibitors retained a preserved microtubule cytoskeleton in the face of disruptive stimuli, indicative of enhanced microtubule stabilization. Second, during HGF-induced migration, TNKS/2-inhibited cells experienced delayed recruitment of APC at the leading edge. Third, as a consequence of all these interferences, TNKS/2-inhibited cells showed deteriorated orientation of the centrosome towards the leading edge. Cell migration relies on sequential waves of protrusive activities at the leading edge. Therefore, our observation that membrane projections and APC cortical targeting were deferred in treated cells can well explain the negative impact of tankyrase inhibition on cell motility as a whole.

The identification of TNKS/2 effectors responsible for the observed activities on cell polarity and directional migration will likely prove daunting. The manifold outcomes of TNKS/2 inhibition, which appear to vary in different cellular settings and at different moments of the cell life cycle, illustrate the versatile nature of such enzymes, which in turn is rooted in the plethora of potential substrates and interactors. It is therefore conceivable that TNKS/2 redundantly affect cell migration by influencing the fate and function of many substrates rather than by selective modulation of one partner. Although a more precise elucidation of how such interconnections mechanistically contribute to the anti-migratory effects of TNKS/2 inhibition awaits further studies, our work illuminates new angles in the evolving landscape of tankyrase-related biology and sets the stage for widening the potential scope of TNKS/2-tailored strategies beyond the currently prevailing paradigms.

## Conclusions

Collectively, our results highlight a crucial role for tankyrases in maneuvering the interphase microtubule apparatus at various levels, from dynamic instability to localization of polarity signals. These findings add new layers of information to our current knowledge of tankyrase biology and may inform new approaches for the preclinical and clinical evaluation of anti-TNKS/2 drugs.

## Methods

### Cell cultures, reagents, vectors, and viral infection

A549, H460, H322, HCC827, and DLD1 cells were purchased from ATCC (Manassas, VA) and cultured in Roswell Park Memorial Institute (RPMI) medium (Sigma-Aldrich, Saint Louis, MO). Their genetic identity was validated by short tandem repeat profiling (Cell ID, PromegaFitchburg, WI). Antibodies were rabbit anti-tankyrase-1/2, rabbit anti-APC, and goat anti-actin (Santa Cruz Dallas, TX); rabbit anti-axin1, rabbit anti-phospho-GSK3-β Ser9, and rabbit anti-GSK3β (Cell Signaling Danvers, MA); and mouse anti-α-tubulin and mouse anti γ-tubulin (Sigma). XAV939 and JNJ-BJ were provided by Janssen Pharmaceutica NV. Recombinant human HGF and Wnt3a were purchased from Peprotech (Rocky Hill, NJ) and R&D Systems (Minneapolis, MN), respectively. Lentiviral pLKO.1-puro short hairpin RNA vectors targeting TNKS (Clone ID: TRCN0000040186) and TNKS2 (Clone ID: TRCN0000053239), as well as the non-targeting control vector (product number: SHC002), were purchased from Sigma. Lentiviral vectors were produced by LipofectAMINE 2000 (Invitrogen Carlsbad, CA)-mediated transfection of 293 T cells.

### TOPflash reporter assay

Cells at 80 % confluence were transiently transfected with TOPflash or FOPflash plasmids (Millipore Billerica, MA) using LipofectAMINE 2000. Luciferase activity was assayed 48 or 72 h after transfection with the Luciferase Assay System (Promega), using a GloMax 96 microplate luminometer (Promega).

### Biological assays

Prior to all of the following experimental procedures and if not otherwise specified, cells were pre-incubated with TNKS/2 inhibitors for 24 h in 2 % fetal bovine serum (FBS)-containing medium; RPMI supplemented with 2 % FBS and HGF (50 ng/mL) was used as chemoattractant. Unless stated differently, TNKS/2 inhibitors were used at a 10 μM concentration.

For invasion studies, 1 × 10^5^ cells in 100 μL of serum-deprived RPMI were seeded onto Transwell chamber inserts (Costar Thermo Fisher, Waltham, MA) with 8-μM pore size membranes coated with Matrigel (R&D Systems) (15 μg/cm^2^). HGF-conditioned medium was added to the lower compartment with or without TNKS/2 inhibitors. Cells were allowed to migrate at 37 °C for 24 h. After mechanical removal of the non-invading cells, the proportion of cells that had migrated to the lower side of the membrane were fixed with 11 % glutaraldehyde, stained with 0.1 % crystal violet, and quantified using ImageJ.

For wound healing assays, cell monolayers were scratched with a pipette tip. Alternatively, IBIDI Culture-Inserts (Martinsried, Germany) with a well-defined cell-free gap were used according to the manufacturer’s guidelines. In both cases, wound-edge cells were stimulated with HGF and allowed to migrate for 24 h with or without TNKS/2 inhibitors prior to crystal violet staining and ImageJ quantification.

Proliferative response was assessed as described previously [47]. On day 0, cells were plated at clonal density (10 cells/μL) in complete medium. On day 1, serially diluted TNKS/2 inhibitors (dose range: 0 to 10 μM) or vehicle (dimethyl sulfoxide) were added to the cells. Drug-containing medium was renewed after 48 h. On day 4, cell viability was measured by CellTiter-Glo (Promega) using a Victor X4 microplate luminometer (PerkinElmer Waltham, MA).

### Immunofluorescence, confocal microscopy, and morphometric quantitation

Cells were seeded onto glass coverslips coated with 3 μg/mL fibronectin (Sigma), fixed in 4 % paraformaldehyde for 10 min, permeabilized with ice-cold methanol for 1 min, and incubated with primary antibodies for 1 h at room temperature, followed by a 30-min staining with Alexa Fluor 555-conjugated or 488-conjugated secondary antibodies (Molecular Probes Thermo Fisher). Nuclei were counterstained with 4',6-diamidino-2-phenylindole (Roche Applied Science Penzberg, Germany). Images were acquired by sequential scanning using the Leica TCS SPE confocal system and a DM 5500 Q microscope (Leica Microsystems Wetzlar, Germany) equipped with a 63× objective. For quantification of lamellipodia extensions, the diffuse cytoplasmic fluorescence of APC was used to trace the silhouette of individual cells facing the wound; cells exhibiting discernible membrane projections were scored as lamellipodia-positive. APC-decorated protrusions were counted by dividing the number of cortical APC events by the total number of lamellipodia. To detect polymerized tubulin (i.e., intact microtubules), we used custom-made image analysis algorithms written in MATLAB. Images were filtered with a linear rotating kernel filter [48] and then processed with a multiscale vessel enhancement filter [49]. To quantify the differences in tubulin staining distribution and morphology we measured the total area occupied by microtubules (a measure of the amount of polymerized tubulin) relative to the total cell area.

### Time-lapse videomicroscopy

Confluent A549 cells were seeded on glass bottom dishes (Willcowells Amsterdam, The Netherlands), scratched with a pipette tip, and treated with HGF with or without TNKS/2 inhibitors. Phase-contrast images were taken every 12 s for 60 min with a 20× objective using a Leica AF6000LX workstation equipped with a thermostatic and CO_2_-controlled chamber. Movies were generated by the LAS AF Leica Application Suite software (Leica) and compressed to 20 frames per second.

### Real Time RT-PCR

Total RNA was extracted with the RNeasy Mini Kit (QiagenHilden, Germany) and reverse-transcribed using High Capacity cDNA reverse transcription (Life Technologies Carlsbad, CA). Results were normalized to the average of three housekeeper genes. The TaqMan probes (Life Technologies) were as follows: Hs99999903_m1 (ACTB), Hs00427621_m1* (TBP), Hs00824723_m1* (UBC), Hs00186671_m1 (TNKS), Hs00228829_m1 (TNKS2), Hs00610344_m1* (AXIN2), Hs00173664_m1 (LGR5), Hs00905030_m1* (MYC), Hs00256886_m1* (HOXB9), and Hs01547250_m1* (LEF1).

### Statistical analysis

Statistical analyses were performed by two-tailed Student’s *t*-test, Chi-square test, and two-way analysis of variance. *P* < 0.05 was considered statistically significant.

## Additional files

Additional file 1:
**Synthesis scheme for JNJ-BJ.** (DOCX 80 kb) Additional file 2:
**Raw data for Fig.** 1 **.** (XLSX 41 kb) Additional file 3:
**Raw data for Fig.** 2 **.** (XLSX 128 kb) Additional file 4: Figure S1. TNKS inhibition does not substantially affect cell proliferation. (PPTX 68 kb) Additional file 5:
**Raw data for Fig. S1.** (XLSX 155 kb) Additional file 6: Figure S2. RNA interference-mediated downregulation of TNKS phenocopies pharmacologic inhibition in impairing cell invasion and migration. (PPTX 243 kb) Additional file 7:
**Raw data for Fig. S2.** (XLSX 48 kb) Additional file 8: Figure S3. β-catenin/TCF-dependent readouts are not substantially influenced by TNKS inactivation. (PPTX 69 kb) Additional file 9:
**Raw data for Fig. S3.** (XLSX 161 kb) Additional file 10: Movie M1. Inactivation of TNKS weakens protrusive activity and lamellipodia formation. (AVI 20080 kb) Additional file 11;
**Raw data for Fig.** 3 **.** (XLSX 37 kb) Additional file 12: Figure S4. The microtubule network is stabilized by TNKS pharmacologic neutralization. (PPTX 1715 kb) Additional file 13:
**Raw data for Fig.** 4. (XLSX 15 kb) Additional file 14: Figure S5. TNKS silencing stabilizes the microtubule network. (PPTX 687 kb) Additional file 15:
**Raw data for Fig.** 5 **.** (XLSX 47 kb) Additional file 16: Figure S6. Centrosome relocation is perturbed by TNKS silencing. (PPTX 239 kb) Additional file 17:
**Raw data for Fig.** 6 **.** (XLSX 35 kb) Additional file 18: Figure S7. TNKS silencing prevents the reallocation of APC at cortical sites. (PPTX 354 kb) Additional file 19: Figure S8. TNKS is recruited to cortical areas in migrating cells. (PPTX 2189 kb)
