# Supplementary material for: Tankyrase inhibition impairs directional migration and invasion of lung cancer cells by affecting microtubule dynamics and polarity signals
Source: BMC Biol. 2016 Jan 19;14:5. doi: 10.1186/s12915-016-0226-9 (PMC4719581; doi:10.1186/s12915-016-0226-9)
Supplement: Additional file 1: — Synthesis scheme for JNJ-BJ. (DOCX 80 kb) [file 12915_2016_226_MOESM1_ESM.docx]

**Tankyrase inhibition impairs directional migration and invasion**

**of lung cancer cells by affecting microtubule dynamics and polarity signals**

Barbara Lupo, Jorge Vialard, Patrick Angibaud, Francesco Sassi, Emanuela Pupo,

Letizia Lanzetti, Paolo M. Comoglio, Andrea Bertotti, and Livio Trusolino

**Supplementary information: Additional File 1**

**Synthesis scheme for JNJ-BJ**

|  | a) Preparation of 7-bromo-3-ethyl-2-methoxy-quinoline |  |
| --- | --- | --- |

Methanol, sodium salt (30% in MeOH)(0.924 mol) was added dropwise at room temperature to a solution of 7-bromo-2-chloro-3-ethyl-quinoline (0.185 mol) in MeOH (500ml). The mixture was stirred and refluxed for 15 hours and poured out into ice water and extracted with DCM. The organic layer was dried (MgSO_4_), filtered and the solvent was evaporated. The residue was purified by column chromatography over silica gel (eluent: DCM 100%). The pure fractions were collected and the solvent was evaporated, yielding 44.6 g (91%) of 7-bromo-3-ethyl-2-methoxy-quinoline.

^1^H NMR (400 MHz, DMSO-d_6_) δ 8.04 (s, 1H), 7.92 (s, 1H), 7.80 (d, *J*=8.5 Hz, 1H), 7.55 (dd, *J*=1.8, 8.5 Hz, 1H), 4.0 (s, 3H), 2.67 (q, *J*=7.4 Hz, 2H), 1.23 (t, *J*=7.4 Hz, 3H).

|  | b) Preparation of 1-(3-ethyl-2-methoxy-7-quinolyl)ethanol |  |
| --- | --- | --- |

n-Butyl-lithium (1.6 M in hexane)(0.124 mol) was added dropwise at -78°C to a solution of intermediate 1 (0.113 mol) in dry THF (300ml) under N_2_ flow. The mixture was stirred at -78°C for 1 hour. A solution of acetaldehyde (0.225 mol) in dry THF (30 mL) was added. The mixture was stirred at -78°C for 2 hours, poured out into ice water + NH_4_Cl and extracted with EtOAc. The organic layer was dried (MgSO_4_), filtered and the solvent was evaporated. The residue was purified by column chromatography over silica gel (elution gradient: DCM/MeOH from 100/0 to 98/2). The pure fractions were collected and the solvent was evaporated, yielding 25.1 g (96%) of 1-(3-ethyl-2-methoxy-7-quinolyl)ethanol.

^1^H NMR (400 MHz, DMSO-d_6_) δ 7.98 (s, 1H), 7.77 (d, *J*=8.6 Hz, 1H), 7.71 (s, 1H), 7.40 (dd, *J*=1.7, 8.6 Hz, 1H), 5.28 (d, *J*=7.4 Hz, 1H) 4.88 (quint., *J*=7.4 Hz, 1H), 4.02 (s, 3H), 2.68 (q, *J*=7.6 Hz, 2H), 1.41 (d, *J*=7.4 Hz, 3H), 1.35 (t, *J*=7.6 Hz, 3H).

|  | c) Preparation of 1-(3-ethyl-2-methoxy-7-quinolyl)ethanone |  |
| --- | --- | --- |

A mixture of 1-(3-ethyl-2-methoxy-7-quinolyl)ethanol (0.05 mol) and manganese oxide (0.133 mol) in 1,4-dioxane (200 mL) was stirred at reflux for 5 hours. After filtration over celite, the filtrate was evaporated, yielding 10.36 g (90%) of 1-(3-ethyl-2-methoxy-7-quinolyl)ethanone, melting point 98°C.

^1^H NMR (400 MHz, DMSO-d_6_) δ 8.35 (s, 1H), 8.11 (s, 1H), 7.86-7.96 (m, 2H), 4.07 (s, 3H), 2.68-2.76 (m, 5H), 1.26 (t, *J*=7.6 Hz, 3H).

|  | d) Preparation of 2-(3-ethyl-2-methoxy-7-quinolyl)propanenitrile |  |
| --- | --- | --- |

2-methyl-2-propanol, potassium salt (0.162 mol) was added portionwise at 10°C to a solution of tosylmethyl isocyanide (0.081 mol) in DMSO (90 ml) and MeOH (8ml). The mixture was stirred for 15 minutes. 1-(3-ethyl-2-methoxy-7-quinolyl)ethanone (0.0353 mol) was added. The mixture was stirred at 10°C for 1.5 hours, poured in ice-water and extracted with EtOAc. The organic layer was dried (MgSO_4_), filtered and the solvent was evaporated. This fraction was purified by column chromatography over silica gel (elution gradient: DCM/MeOH from 100/0 to 98/2). The pure fractions were collected and the solvent was evaporated, yielding 7.88 g (93%) of 2-(3-ethyl-2-methoxy-7-quinolyl)propanenitrile.

^1^H NMR (400 MHz, DMSO-d_6_) δ 8.03 (s, 1H), 7.88 (d, *J*=8.6 Hz, 1H), 7.78 (s, 1H), 7.44 (dd, *J*=1.8, 8.6 Hz, 1H), 4.48 (q, *J*=7.4 Hz, 1H), 4.03 (s, 3H), 2.68 (q, *J*=7.6 Hz, 2H), 1.64 (d, *J*=7.4 Hz, 3H), 1.25 (t, *J*=7.6 Hz, 3H).

|  | Preparation of 3-[2-cyano-2-(3-ethyl-2-oxo-1H-quinolin-7-yl)propyl]benzonitrile |  |
| --- | --- | --- |

2-methyl-2-propanol, potassium (0.0008 mol) was added portionwise at 5°C to a solution of 2-(3-ethyl-2-methoxy-7-quinolyl)propanenitrile (39003900-AAA) (0.0004 mol) and 3-(bromomethyl)- benzonitrile (0.0012 mol) in THF (2ml) under N_2_ flow. The mixture was stirred at 5°C for 1 hour, then stirred at room temperature for 1 hour and poured out into ice water. EtOAc was added. The mixture was extracted with EtOAc. The organic layer was washed with saturated NaCl, dried (MgSO_4_), filtered and the solvent was evaporated. The residue was purified by column chromatography over silica gel (3.5μm) (eluent: DCM 100). The pure fractions were collected and the solvent was evaporated, yielding 0.146g (99%) of 3-[2-cyano-2-(3-ethyl-2-oxo-1H-quinolin-7-yl)propyl]benzonitrile.

^1^H NMR (400 MHz, DMSO-d_6_) δ 8.05 (s, 1H), 7.92 (d, *J*=8.6 Hz, 1H), 7.76 (s, 1H), 7.71 (d, *J*=7.6 Hz, 1H), 7.53-7.60 (m, 2H), 7.42-7.51 (m, 1H), 7.35-7.41 (m, 1H), 4.02 (s, 3H), 3.43 (s, 2H), 2.70 (q, *J*=7.3 Hz, 2H), 1.81 (s, 3H), 1.21-1.29 (t, *J*=7.3 Hz, 3H).

|  | Preparation of (R or S)-3-[2-cyano-2-(3-ethyl-2-oxo-1H-quinolin-7-yl)propyl]benzonitrile |  |
| --- | --- | --- |

HCl 3N (1ml) was added dropwise at room temperature to a solution of a racemic mixture of 3-[2-cyano-2-(3-ethyl-2-oxo-1H-quinolin-7-yl)propyl]benzonitrile (39258609-AAA) (0.0004 mol) in dioxane (2ml). The mixture was stirred at 80°C overnight, then cooled to room temperature, poured out into ice water. EtOAc was added. The mixture was basified with potassium carbonate and extracted with EtOAc. The organic layer was separated, dried (MgSO_4_), filtered and the solvent was evaporated. The residue(0.09g, 67%) was washed with diethyl ether. The precipitate was filtered off and dried under vacuo, yielding 0.067g (50%) of 3-[2-cyano-2-(3-ethyl-2-oxo-1H-quinolin-7-yl)propyl]benzonitrile, melting point: 192°C.

^1^H NMR (400 MHz, DMSO-d_6_) δ 11.72 (s, 1H), 7.74-7.69 (m, 3H), 7.53 (s, 1H), 7.48 (t, *J*= 7.8 Hz, 1H), 7.35-7.29 (m, 3H), 3.35 (m, 2H), 2.5 (s, 2H), 1.75 (s, 3H), 1.18 (t, *J*=7.6 Hz, 3H).

Enantiomers were separated by chromatography over silica gel [Chiralpak® AD (20μm) (eluent: MeOH 100%)]. First eluted enantiomer fractions were collected and the solvent was evaporated, yielding: 0.26 g of Fraction 1. Fraction 1 was triturated in Et_2_O, the precipitate was filtered off and dried under vacuo, yielding 0.225g (33%) of (R or S)-3-[2-cyano-2-(3-ethyl-2-oxo-1H-quinolin-7-yl)propyl]benzonitrile , melting point: 196°C and [α]_D_ ^20^ = +90.3 (DMF; c=0.5 mg/ml).

^1^H NMR (400 MHz, DMSO-d_6_) δ 11.72 (s, 1H), 7.75-7.68 (m, 3H), 7.52 (s, 1H), 7.49 (t, *J*= 7.8 Hz, 1H), 7.37-7.30 (m, 3H), 3.35 (s, 2H), 2.5 (s, 2H), 1.75 (s, 3H), 1.19 (t, *J*=7.6 Hz, 3H).
