# Supplementary material for: Tankyrase inhibition impairs directional migration and invasion of lung cancer cells by affecting microtubule dynamics and polarity signals
Source: BMC Biol. 2016 Jan 19;14:5. doi: 10.1186/s12915-016-0226-9 (PMC4719581; doi:10.1186/s12915-016-0226-9)
Supplement: Additional file 4: Figure S1. — TNKS inhibition does not substantially affect cell proliferation. (PPTX 68 kb) [file 12915_2016_226_MOESM4_ESM.pptx]

## Slide 1
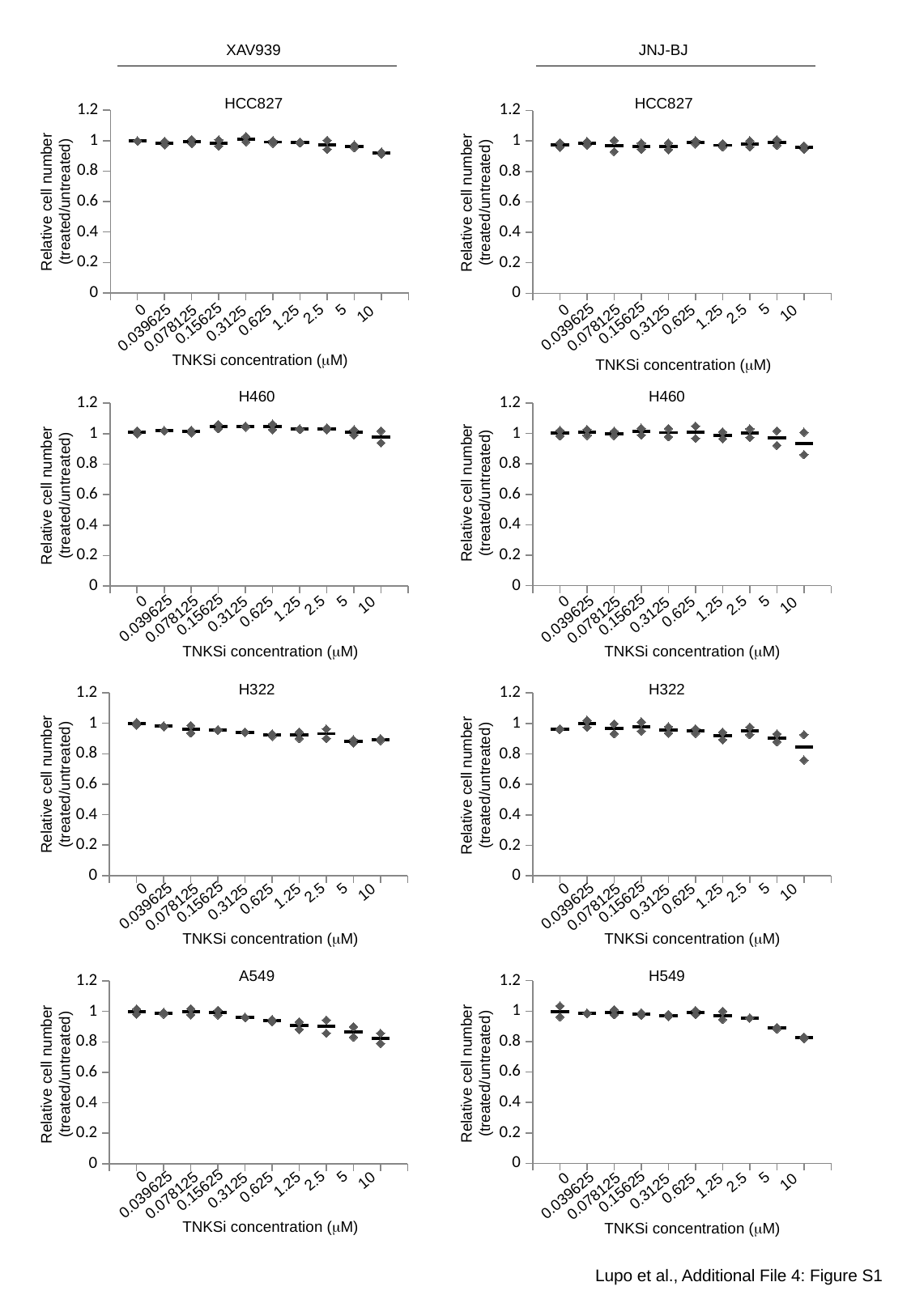

XAV939
XAV939
JNJ-BJ
HCC827
HCC827
### Chart
| Category | XAV939 Average 1 | XAV939 Average 2 | XAV939 |
|---|---|---|---|
### Chart
| Category | JNJ-BJ Average 1 | JNJ-BJ Average 2 | JNJ-BJ |
|---|---|---|---|Relative cell number
(treated/untreated)
Relative cell number
(treated/untreated)
5
0
5
0
10
2.5
10
2.5
1.25
1.25
0.625
0.625
0.15625
0.3125
0.15625
0.3125
0.039625
0.039625
0.078125
0.078125
TNKSi concentration (mM)
TNKSi concentration (mM)
H460
H460
### Chart
| Category | JNJ-BJ Average 1 | JNJ-BJ Average 2 | JNJ-BJ |
|---|---|---|---|
### Chart
| Category | XAV939 Average 1 | XAV939 Average 2 | XAV939 |
|---|---|---|---|Relative cell number
(treated/untreated)
Relative cell number
(treated/untreated)
5
0
5
0
10
2.5
10
2.5
1.25
1.25
0.625
0.625
0.15625
0.3125
0.15625
0.3125
0.039625
0.039625
0.078125
0.078125
TNKSi concentration (mM)
TNKSi concentration (mM)
H322
H322
### Chart
| Category | XAV939 Average 1 | XAV939 Average 2 | XAV939 |
|---|---|---|---|
### Chart
| Category | JNJ-BJ Average 1 | JNJ-BJ Average 2 | JNJ-BJ |
|---|---|---|---|Relative cell number
(treated/untreated)
Relative cell number
(treated/untreated)
5
5
0
0
10
10
2.5
2.5
1.25
1.25
0.625
0.625
0.15625
0.3125
0.15625
0.3125
0.039625
0.039625
0.078125
0.078125
TNKSi concentration (mM)
TNKSi concentration (mM)
A549
H549
### Chart
| Category | JNJ-BJ Average 1 | JNJ-BJ Average 2 | JNJ-BJ |
|---|---|---|---|
### Chart
| Category | XAV939 Average 1 | XAV939 Average 2 | XAV939 |
|---|---|---|---|Relative cell number
(treated/untreated)
Relative cell number
(treated/untreated)
5
0
5
0
10
2.5
10
2.5
1.25
1.25
0.625
0.625
0.15625
0.3125
0.15625
0.3125
0.039625
0.078125
0.039625
0.078125
TNKSi concentration (mM)
TNKSi concentration (mM)
Lupo et al., Additional File 4: Figure S1

## Slide 2
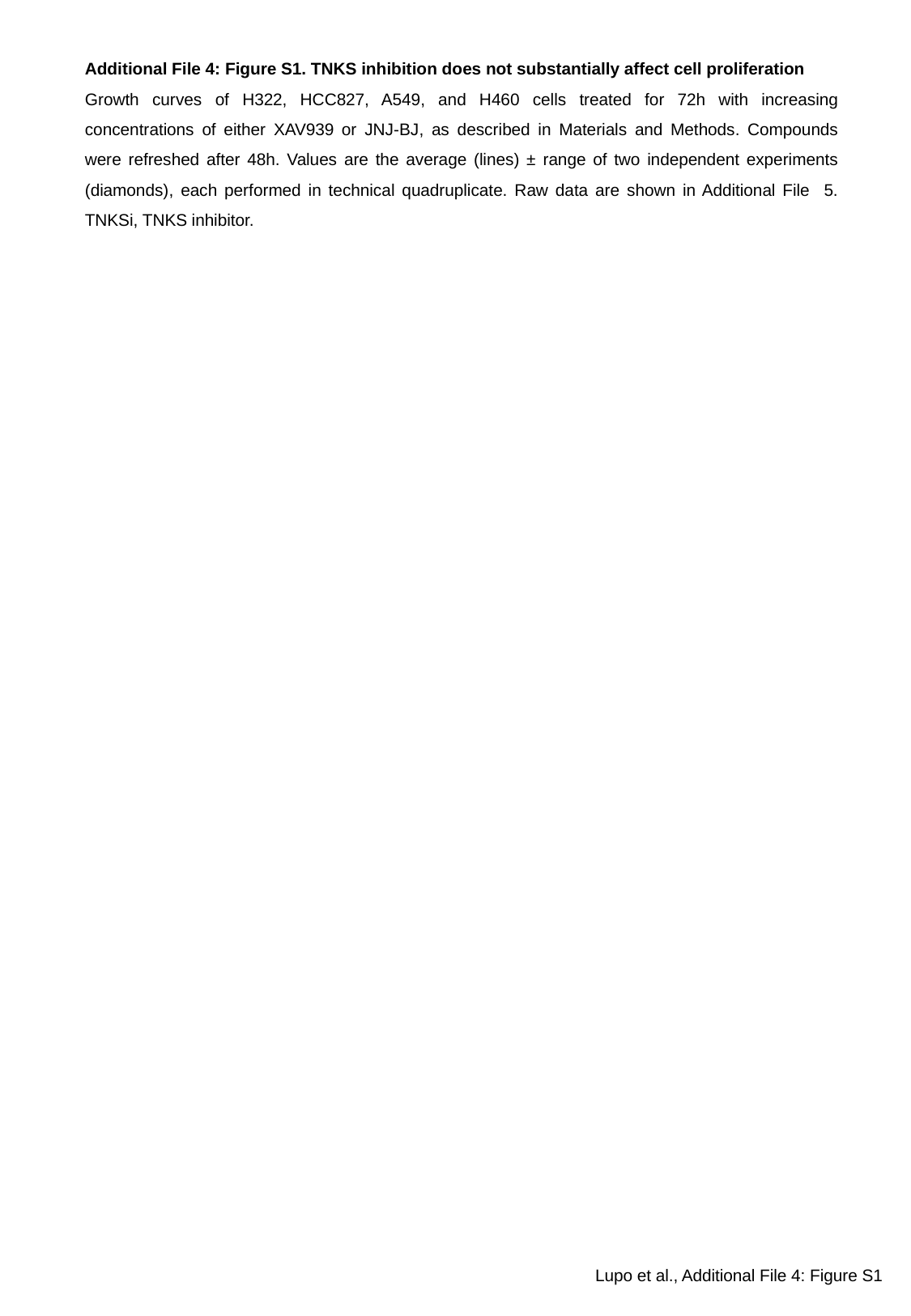

Additional File 4: Figure S1. TNKS inhibition does not substantially affect cell proliferation
Growth curves of H322, HCC827, A549, and H460 cells treated for 72h with increasing concentrations of either XAV939 or JNJ-BJ, as described in Materials and Methods. Compounds were refreshed after 48h. Values are the average (lines) ± range of two independent experiments (diamonds), each performed in technical quadruplicate. Raw data are shown in Additional File 5. TNKSi, TNKS inhibitor.
Lupo et al., Additional File 4: Figure S1
