# Supplementary material for: Tankyrase inhibition impairs directional migration and invasion of lung cancer cells by affecting microtubule dynamics and polarity signals
Source: BMC Biol. 2016 Jan 19;14:5. doi: 10.1186/s12915-016-0226-9 (PMC4719581; doi:10.1186/s12915-016-0226-9)
Supplement: Additional file 6: Figure S2. — RNA interference-mediated downregulation of TNKS phenocopies pharmacologic inhibition in impairing cell invasion and migration. (PPTX 243 kb) [file 12915_2016_226_MOESM6_ESM.pptx]

## Slide 1
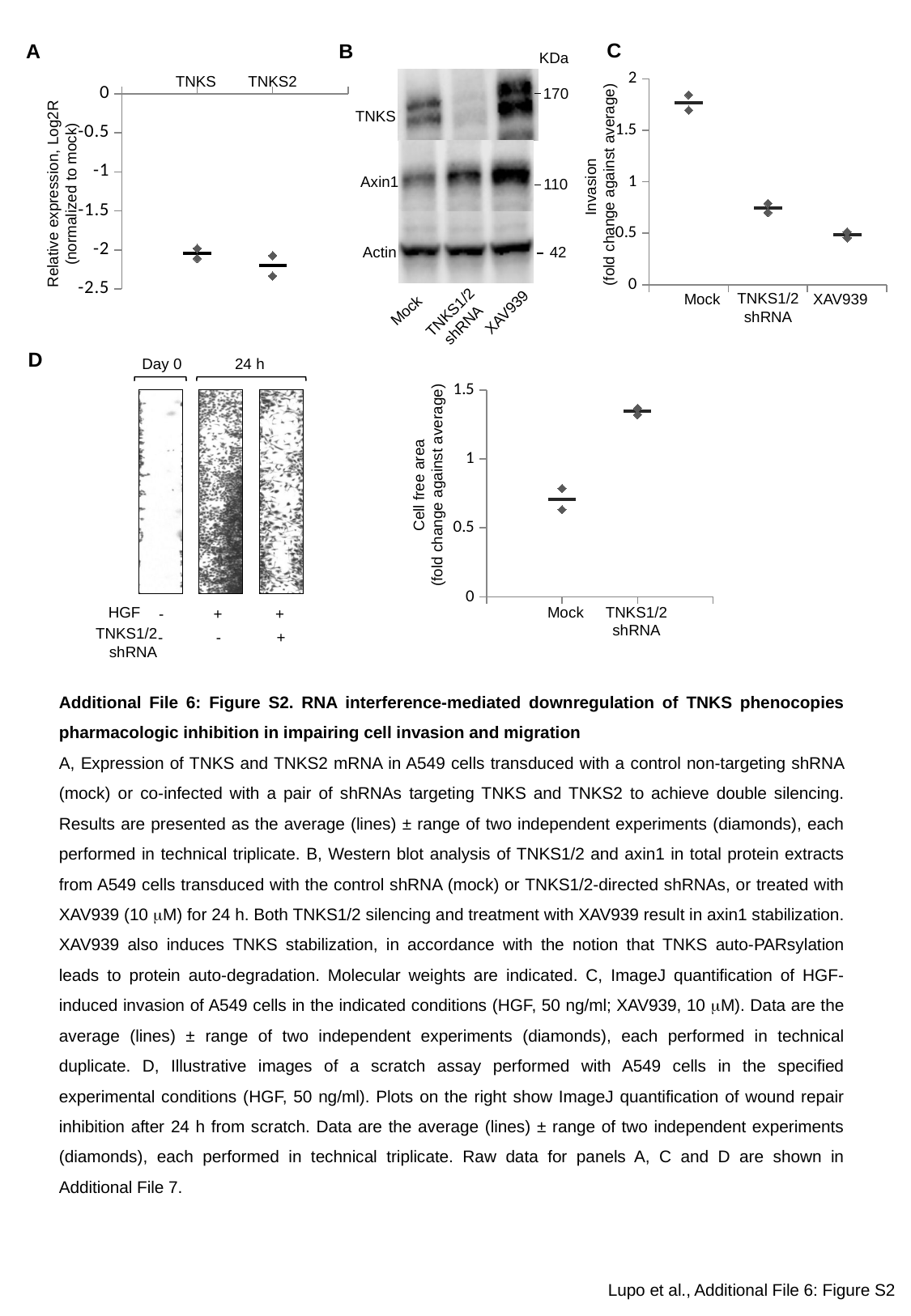

C
A
B
KDa
TNKS
TNKS2
### Chart
| Category | Average 1 | Average 2 | Average 1+2 |
|---|---|---|---|
170
### Chart
| Category | | | |
|---|---|---|---|TNKS
Invasion
(fold change against average)
Axin1
110
Relative expression, Log2R
(normalized to mock)
42
Actin
TNKS1/2
shRNA
XAV939
Mock
TNKS1/2
shRNA
Mock
XAV939
D
Day 0
24 h
### Chart
| Category | Average 1st exp | Average 2nd exp | Average 1+2 exp |
|---|---|---|---|Cell free area
(fold change against average)
TNKS1/2
shRNA
Mock
HGF
-
+
+
TNKS1/2
shRNA
-
-
+
Additional File 6: Figure S2. RNA interference-mediated downregulation of TNKS phenocopies pharmacologic inhibition in impairing cell invasion and migration
A, Expression of TNKS and TNKS2 mRNA in A549 cells transduced with a control non-targeting shRNA (mock) or co-infected with a pair of shRNAs targeting TNKS and TNKS2 to achieve double silencing. Results are presented as the average (lines) ± range of two independent experiments (diamonds), each performed in technical triplicate. B, Western blot analysis of TNKS1/2 and axin1 in total protein extracts from A549 cells transduced with the control shRNA (mock) or TNKS1/2-directed shRNAs, or treated with XAV939 (10 mM) for 24 h. Both TNKS1/2 silencing and treatment with XAV939 result in axin1 stabilization. XAV939 also induces TNKS stabilization, in accordance with the notion that TNKS auto-PARsylation leads to protein auto-degradation. Molecular weights are indicated. C, ImageJ quantification of HGF-induced invasion of A549 cells in the indicated conditions (HGF, 50 ng/ml; XAV939, 10 μM). Data are the average (lines) ± range of two independent experiments (diamonds), each performed in technical duplicate. D, Illustrative images of a scratch assay performed with A549 cells in the specified experimental conditions (HGF, 50 ng/ml). Plots on the right show ImageJ quantification of wound repair inhibition after 24 h from scratch. Data are the average (lines) ± range of two independent experiments (diamonds), each performed in technical triplicate. Raw data for panels A, C and D are shown in Additional File 7.
Lupo et al., Additional File 6: Figure S2
