# Supplementary material for: Tankyrase inhibition impairs directional migration and invasion of lung cancer cells by affecting microtubule dynamics and polarity signals
Source: BMC Biol. 2016 Jan 19;14:5. doi: 10.1186/s12915-016-0226-9 (PMC4719581; doi:10.1186/s12915-016-0226-9)
Supplement: Additional file 8: Figure S3. — β-catenin/TCF-dependent readouts are not substantially influenced by TNKS inactivation. (PPTX 69 kb) [file 12915_2016_226_MOESM8_ESM.pptx]

## Slide 1
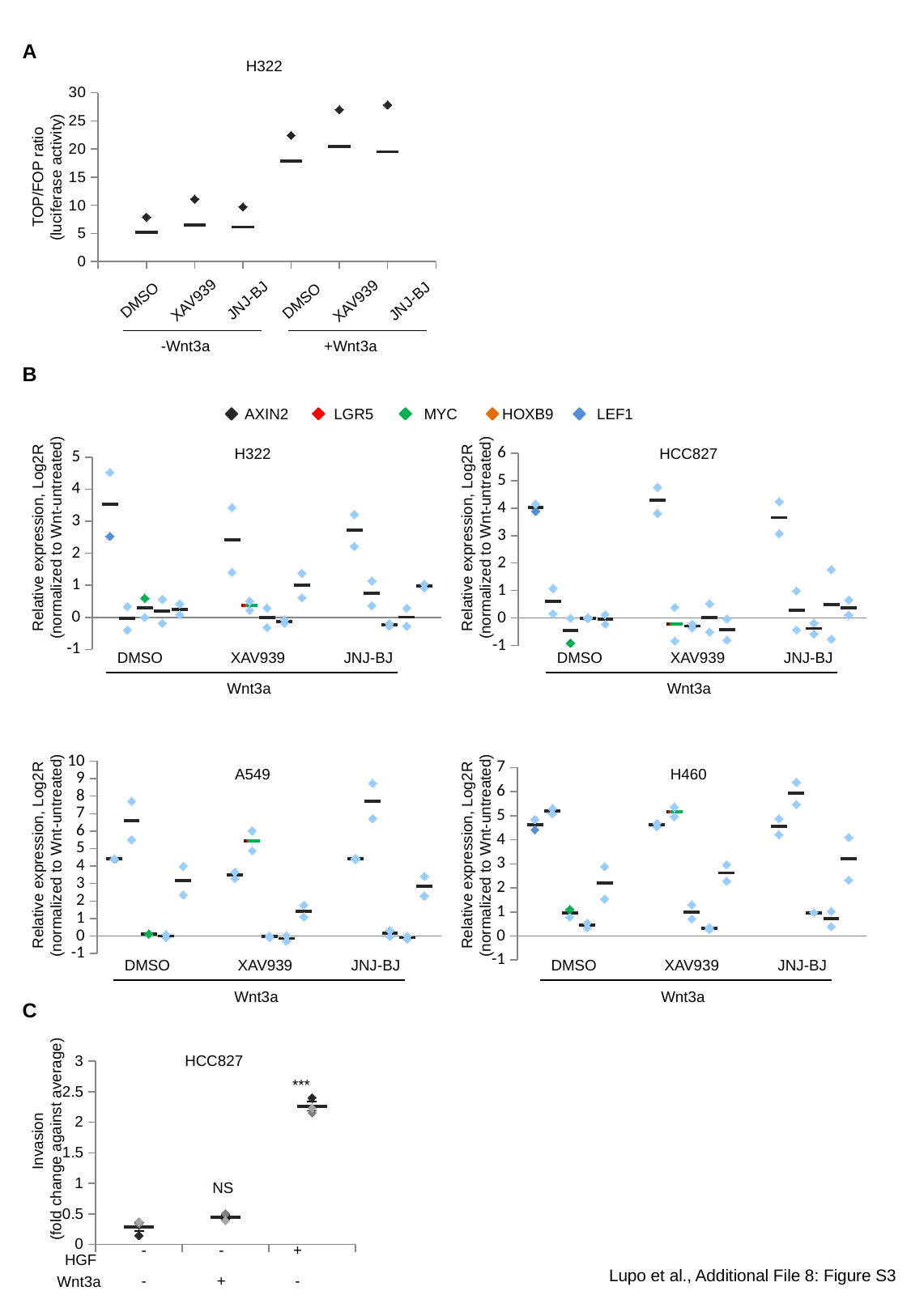

A
H322
### Chart
| Category | | | | |
|---|---|---|---|---|TOP/FOP ratio
(luciferase activity)
DMSO
XAV939
JNJ-BJ
DMSO
XAV939
JNJ-BJ
-Wnt3a
+Wnt3a
B
AXIN2
LGR5
MYC
HOXB9
LEF1
H322
HCC827
### Chart
| Category | | | |
|---|---|---|---|
### Chart
| Category | ΔΔCt 1 | ΔΔCt 2 | ΔΔCt Average 1+2 |
|---|---|---|---|Relative expression, Log2R
(normalized to Wnt-untreated)
Relative expression, Log2R
(normalized to Wnt-untreated)
DMSO
XAV939
JNJ-BJ
DMSO
XAV939
JNJ-BJ
Wnt3a
Wnt3a
### Chart
| Category | | | |
|---|---|---|---|
### Chart
| Category | | | |
|---|---|---|---|A549
H460
Relative expression, Log2R
(normalized to Wnt-untreated)
Relative expression, Log2R
(normalized to Wnt-untreated)
DMSO
XAV939
JNJ-BJ
DMSO
XAV939
JNJ-BJ
Wnt3a
Wnt3a
C
HCC827
### Chart
| Category | Average 1 | Average 2 | Average 3 | Average 1+2+3 |
|---|---|---|---|---|***
Invasion
(fold change against average)
NS
-
-
+
HGF
Lupo et al., Additional File 8: Figure S3
-
+
-
Wnt3a

## Slide 2
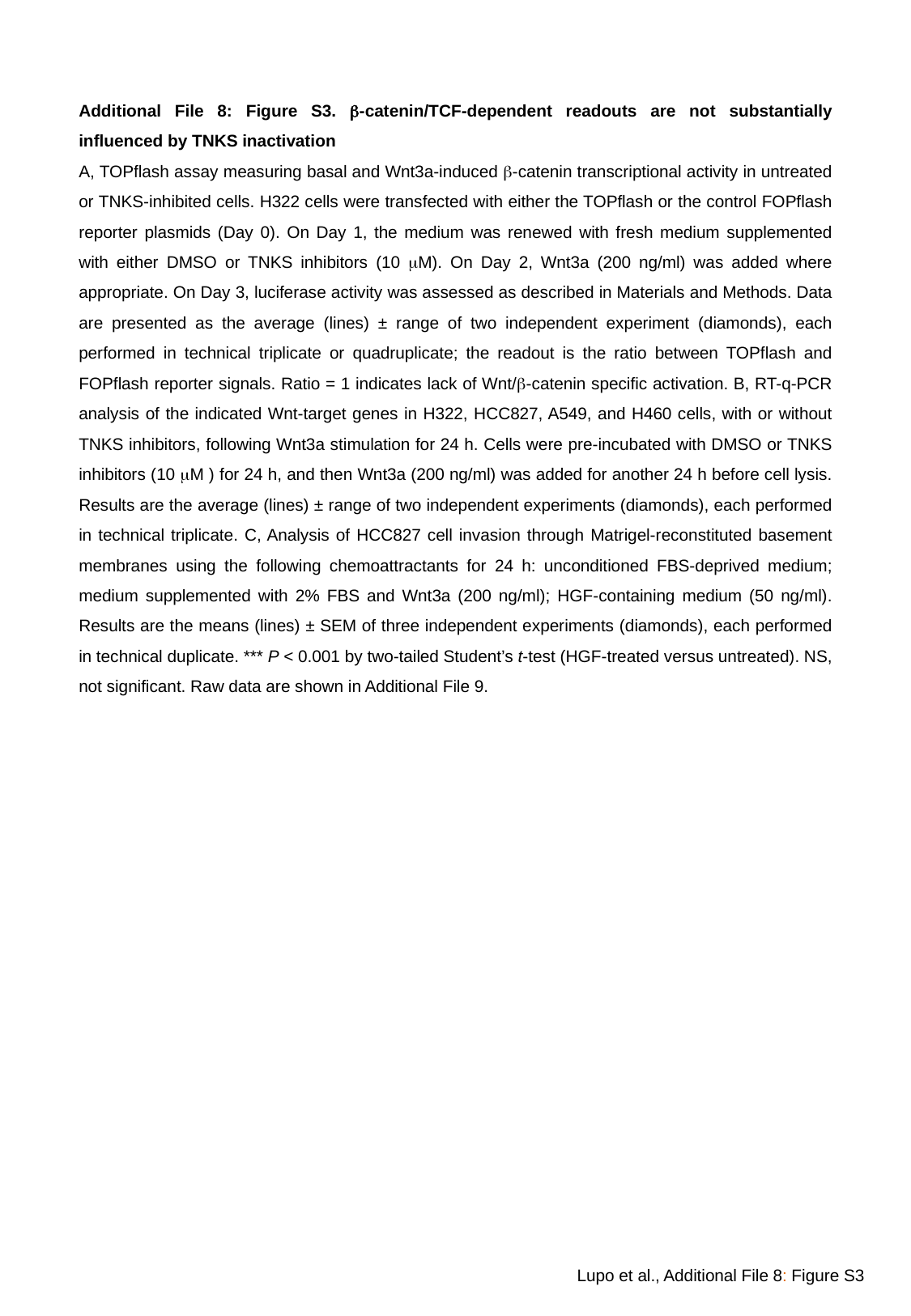

Additional File 8: Figure S3. β-catenin/TCF-dependent readouts are not substantially influenced by TNKS inactivation
A, TOPflash assay measuring basal and Wnt3a-induced β-catenin transcriptional activity in untreated or TNKS-inhibited cells. H322 cells were transfected with either the TOPflash or the control FOPflash reporter plasmids (Day 0). On Day 1, the medium was renewed with fresh medium supplemented with either DMSO or TNKS inhibitors (10 mM). On Day 2, Wnt3a (200 ng/ml) was added where appropriate. On Day 3, luciferase activity was assessed as described in Materials and Methods. Data are presented as the average (lines) ± range of two independent experiment (diamonds), each performed in technical triplicate or quadruplicate; the readout is the ratio between TOPflash and FOPflash reporter signals. Ratio = 1 indicates lack of Wnt/β-catenin specific activation. B, RT-q-PCR analysis of the indicated Wnt-target genes in H322, HCC827, A549, and H460 cells, with or without TNKS inhibitors, following Wnt3a stimulation for 24 h. Cells were pre-incubated with DMSO or TNKS inhibitors (10 mM ) for 24 h, and then Wnt3a (200 ng/ml) was added for another 24 h before cell lysis. Results are the average (lines) ± range of two independent experiments (diamonds), each performed in technical triplicate. C, Analysis of HCC827 cell invasion through Matrigel-reconstituted basement membranes using the following chemoattractants for 24 h: unconditioned FBS-deprived medium; medium supplemented with 2% FBS and Wnt3a (200 ng/ml); HGF-containing medium (50 ng/ml). Results are the means (lines) ± SEM of three independent experiments (diamonds), each performed in technical duplicate. *** P < 0.001 by two-tailed Student’s t-test (HGF-treated versus untreated). NS, not significant. Raw data are shown in Additional File 9.
Lupo et al., Additional File 8: Figure S3
