# Supplementary material for: Tankyrase inhibition impairs directional migration and invasion of lung cancer cells by affecting microtubule dynamics and polarity signals
Source: BMC Biol. 2016 Jan 19;14:5. doi: 10.1186/s12915-016-0226-9 (PMC4719581; doi:10.1186/s12915-016-0226-9)
Supplement: Additional file 12: Figure S4. — The microtubule network is stabilized by TNKS pharmacologic neutralization. (PPTX 1715 kb) [file 12915_2016_226_MOESM12_ESM.pptx]

## Slide 1
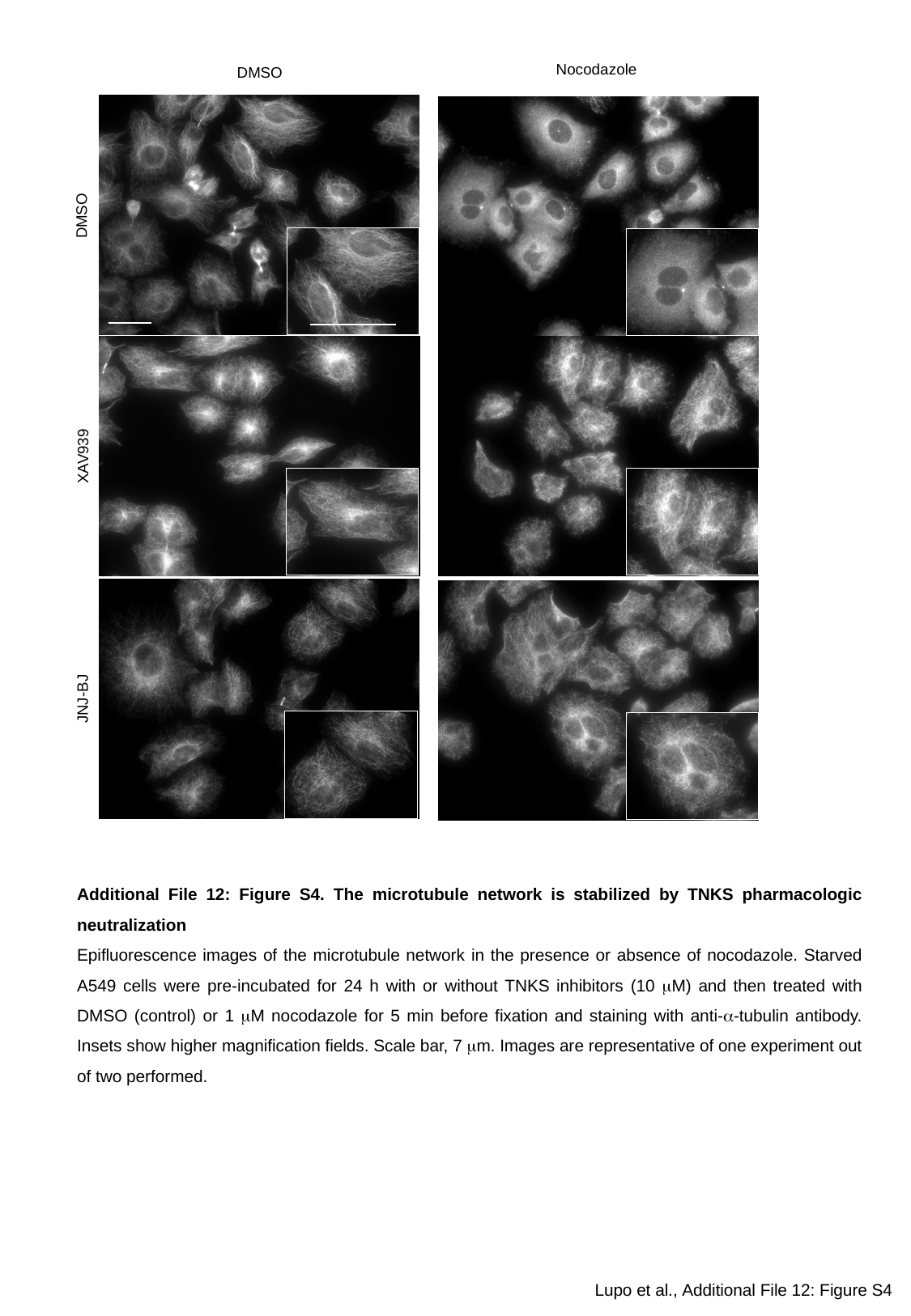

Nocodazole
DMSO
DMSO
XAV939
JNJ-BJ
Additional File 12: Figure S4. The microtubule network is stabilized by TNKS pharmacologic neutralization
Epifluorescence images of the microtubule network in the presence or absence of nocodazole. Starved A549 cells were pre-incubated for 24 h with or without TNKS inhibitors (10 μM) and then treated with DMSO (control) or 1 mM nocodazole for 5 min before fixation and staining with anti-α-tubulin antibody. Insets show higher magnification fields. Scale bar, 7 μm. Images are representative of one experiment out of two performed.
Lupo et al., Additional File 12: Figure S4
