# Supplementary material for: Tankyrase inhibition impairs directional migration and invasion of lung cancer cells by affecting microtubule dynamics and polarity signals
Source: BMC Biol. 2016 Jan 19;14:5. doi: 10.1186/s12915-016-0226-9 (PMC4719581; doi:10.1186/s12915-016-0226-9)
Supplement: Additional file 14: Figure S5. — TNKS silencing stabilizes the microtubule network. (PPTX 687 kb) [file 12915_2016_226_MOESM14_ESM.pptx]

## Slide 1
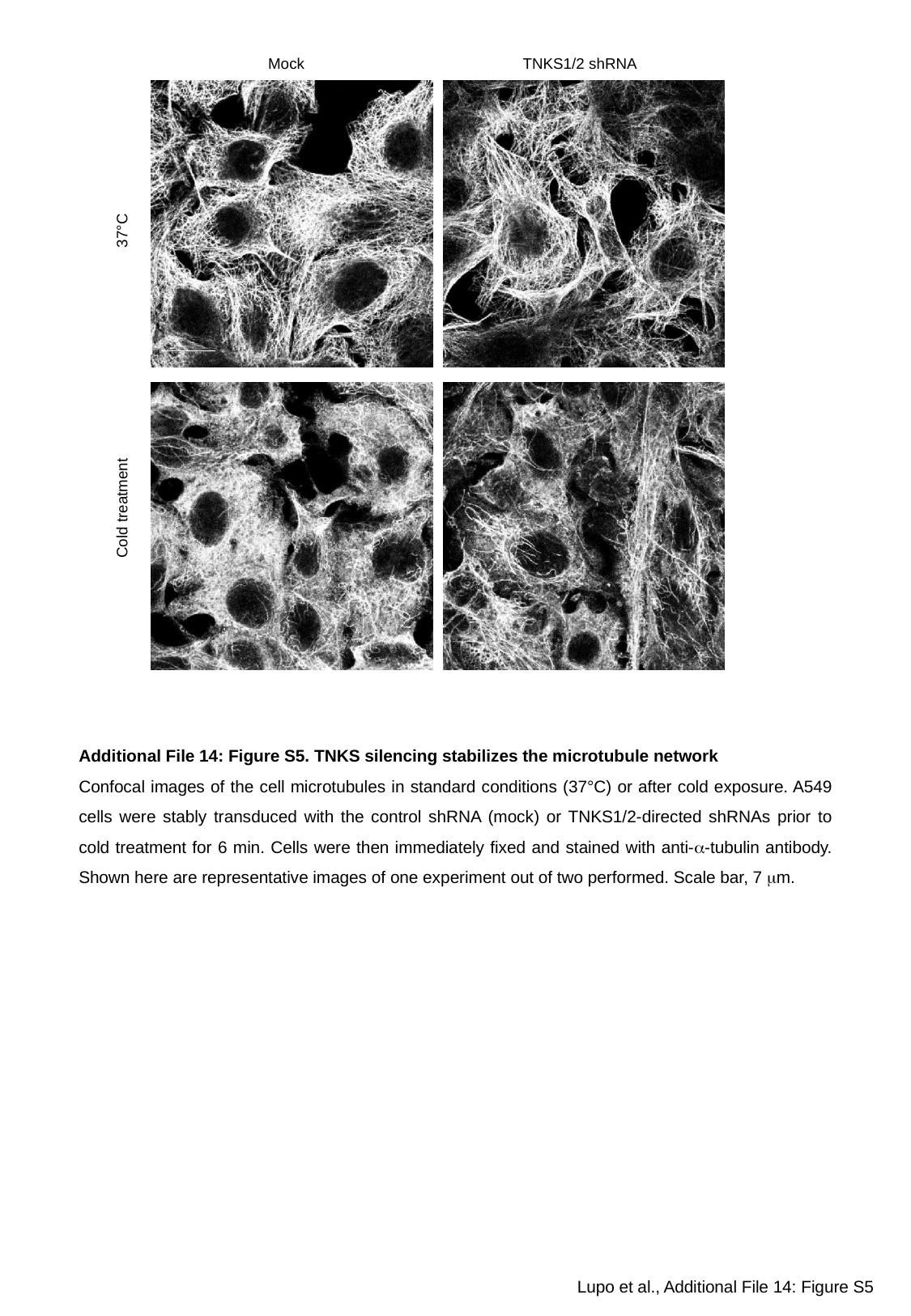

Mock
TNKS1/2 shRNA
37°C
Cold treatment
Additional File 14: Figure S5. TNKS silencing stabilizes the microtubule network
Confocal images of the cell microtubules in standard conditions (37°C) or after cold exposure. A549 cells were stably transduced with the control shRNA (mock) or TNKS1/2-directed shRNAs prior to cold treatment for 6 min. Cells were then immediately fixed and stained with anti-α-tubulin antibody. Shown here are representative images of one experiment out of two performed. Scale bar, 7 mm.
Lupo et al., Additional File 14: Figure S5
