# Supplementary material for: Tankyrase inhibition impairs directional migration and invasion of lung cancer cells by affecting microtubule dynamics and polarity signals
Source: BMC Biol. 2016 Jan 19;14:5. doi: 10.1186/s12915-016-0226-9 (PMC4719581; doi:10.1186/s12915-016-0226-9)
Supplement: Additional file 16: Figure S6. — Centrosome relocation is perturbed by TNKS silencing. (PPTX 239 kb) [file 12915_2016_226_MOESM16_ESM.pptx]

## Slide 1
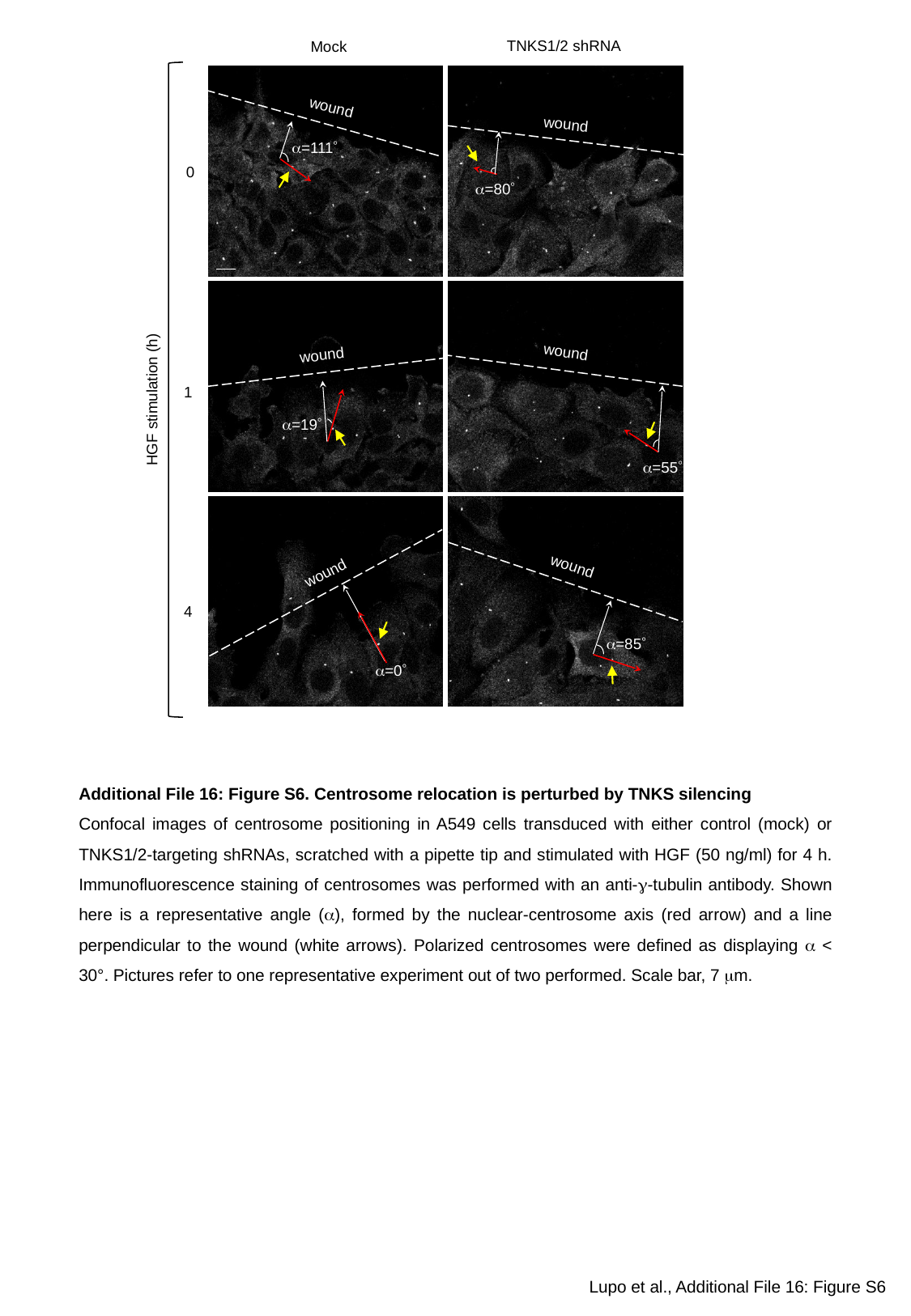

Mock
TNKS1/2 shRNA
wound
wound
wound
a=111°
0
a=80°
wound
wound
1
HGF stimulation (h)
a=19°
a=55°
wound
wound
4
a=85°
a=0°
Additional File 16: Figure S6. Centrosome relocation is perturbed by TNKS silencing
Confocal images of centrosome positioning in A549 cells transduced with either control (mock) or TNKS1/2-targeting shRNAs, scratched with a pipette tip and stimulated with HGF (50 ng/ml) for 4 h. Immunofluorescence staining of centrosomes was performed with an anti-g-tubulin antibody. Shown here is a representative angle (α), formed by the nuclear-centrosome axis (red arrow) and a line perpendicular to the wound (white arrows). Polarized centrosomes were defined as displaying α < 30°. Pictures refer to one representative experiment out of two performed. Scale bar, 7 mm.
Lupo et al., Additional File 16: Figure S6
