# Supplementary material for: Tankyrase inhibition impairs directional migration and invasion of lung cancer cells by affecting microtubule dynamics and polarity signals
Source: BMC Biol. 2016 Jan 19;14:5. doi: 10.1186/s12915-016-0226-9 (PMC4719581; doi:10.1186/s12915-016-0226-9)
Supplement: Additional file 18: Figure S7. — TNKS silencing prevents the reallocation of APC at cortical sites. (PPTX 354 kb) [file 12915_2016_226_MOESM18_ESM.pptx]

## Slide 1
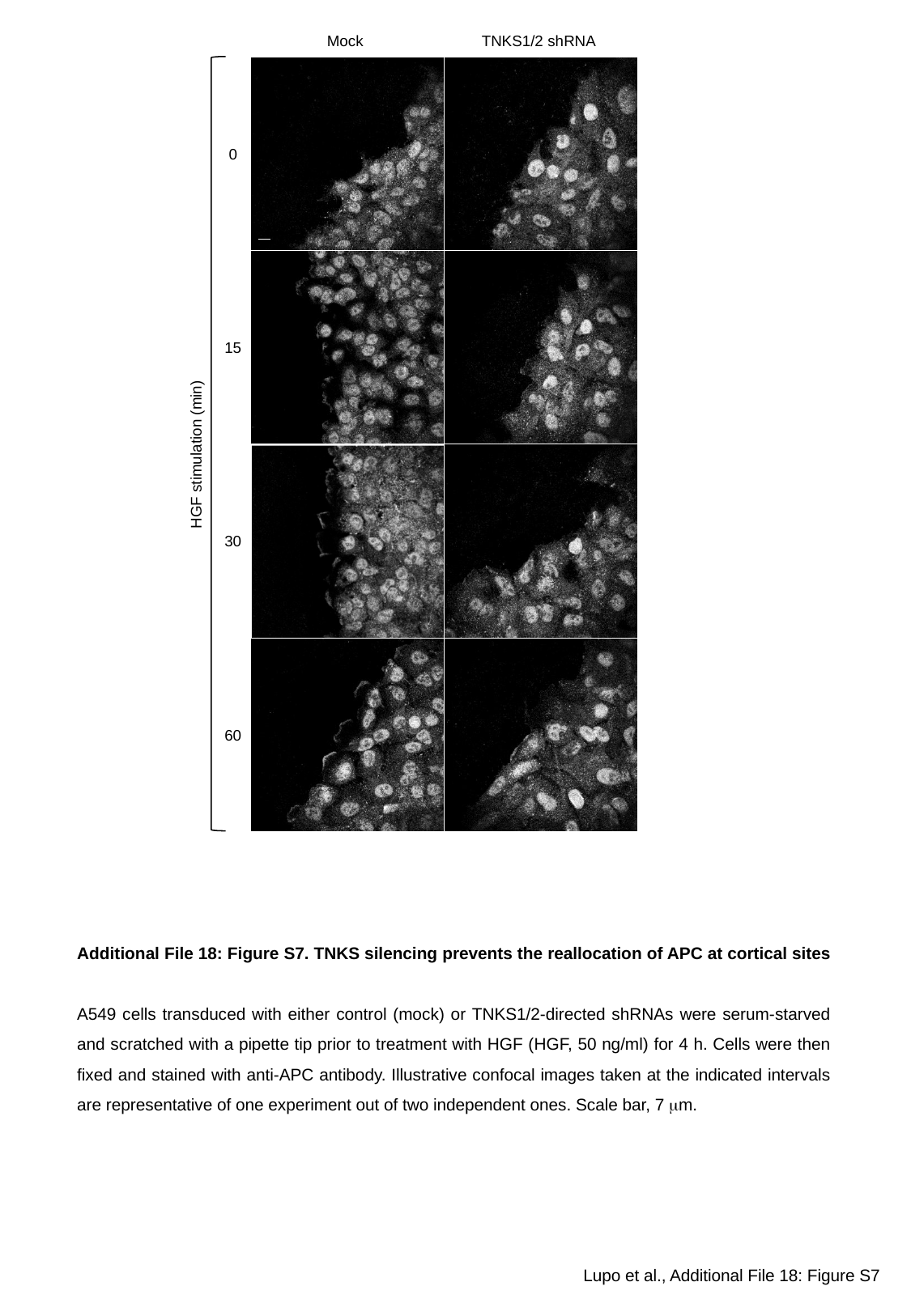

Mock
TNKS1/2 shRNA
0
15
HGF stimulation (min)
30
60
Additional File 18: Figure S7. TNKS silencing prevents the reallocation of APC at cortical sites
A549 cells transduced with either control (mock) or TNKS1/2-directed shRNAs were serum-starved and scratched with a pipette tip prior to treatment with HGF (HGF, 50 ng/ml) for 4 h. Cells were then fixed and stained with anti‑APC antibody. Illustrative confocal images taken at the indicated intervals are representative of one experiment out of two independent ones. Scale bar, 7 mm.
Lupo et al., Additional File 18: Figure S7
