# Supplementary material for: Tankyrase inhibition impairs directional migration and invasion of lung cancer cells by affecting microtubule dynamics and polarity signals
Source: BMC Biol. 2016 Jan 19;14:5. doi: 10.1186/s12915-016-0226-9 (PMC4719581; doi:10.1186/s12915-016-0226-9)
Supplement: Additional file 19: Figure S8. — TNKS is recruited to cortical areas in migrating cells. (PPTX 2189 kb) [file 12915_2016_226_MOESM19_ESM.pptx]

## Slide 1
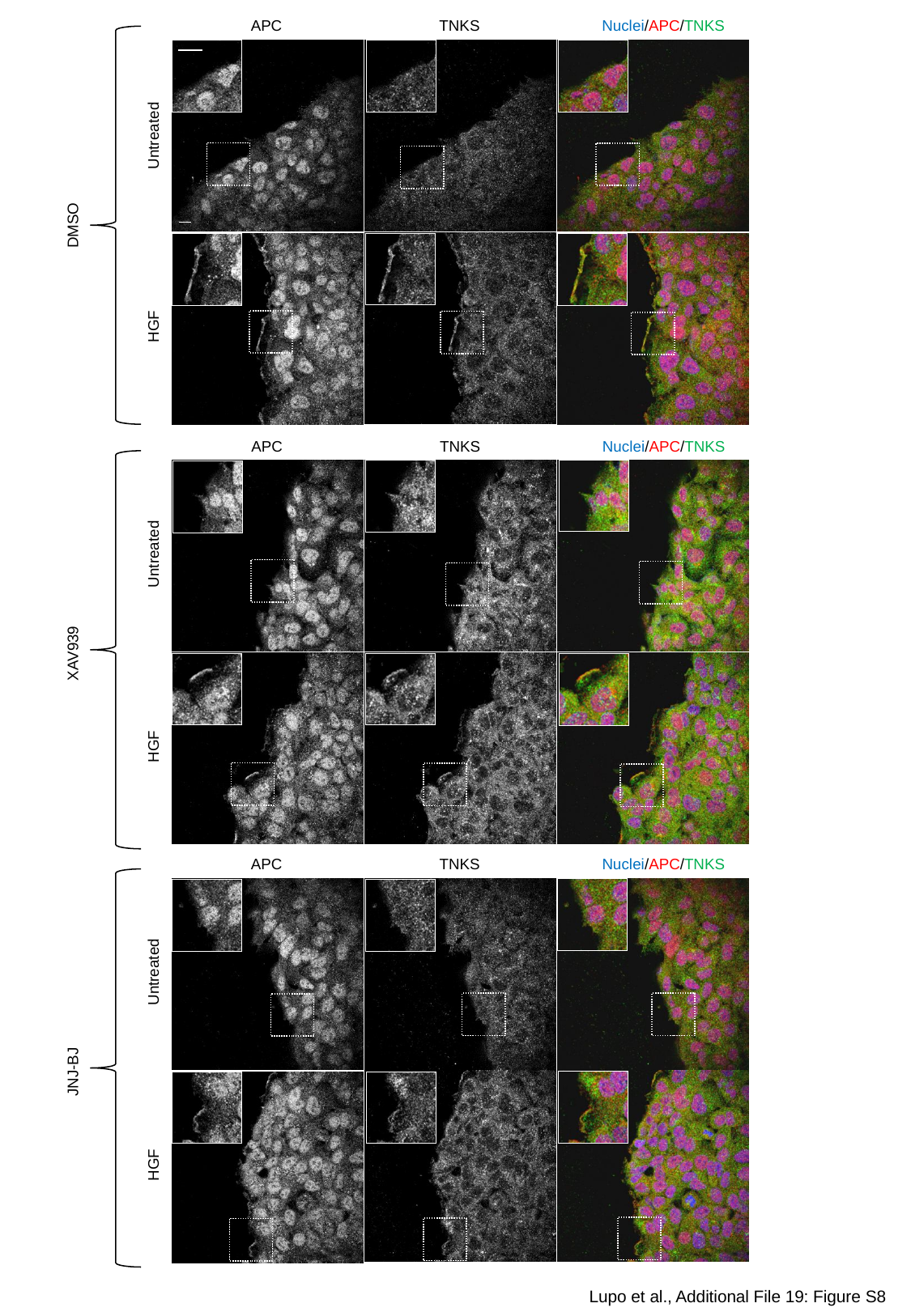

APC
TNKS
Nuclei/APC/TNKS
Untreated
DMSO
 HGF
APC
TNKS
Nuclei/APC/TNKS
Untreated
XAV939
HGF
APC
TNKS
Nuclei/APC/TNKS
Untreated
JNJ-BJ
HGF
Lupo et al., Additional File 19: Figure S8

## Slide 2
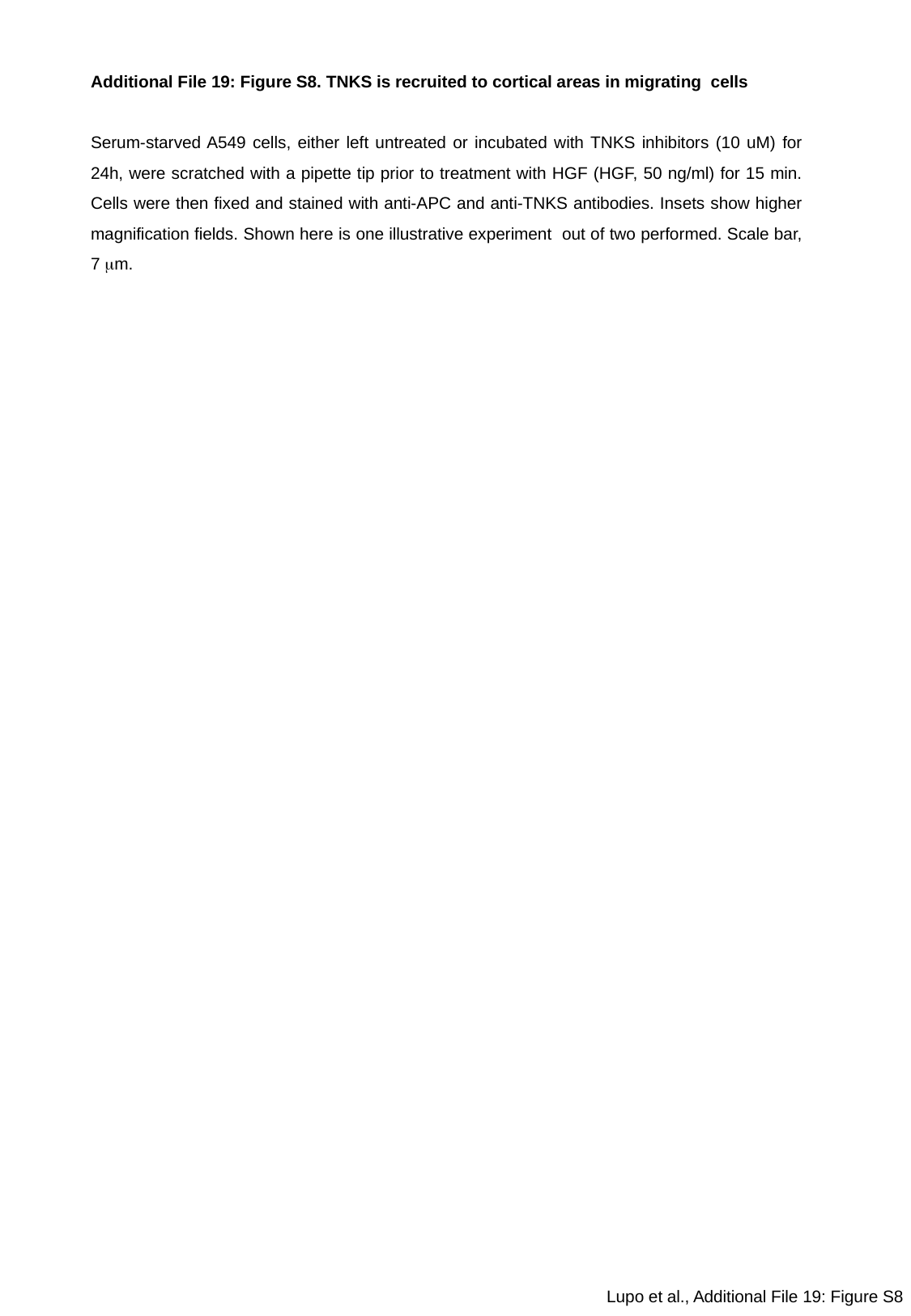

Additional File 19: Figure S8. TNKS is recruited to cortical areas in migrating cells
Serum-starved A549 cells, either left untreated or incubated with TNKS inhibitors (10 uM) for 24h, were scratched with a pipette tip prior to treatment with HGF (HGF, 50 ng/ml) for 15 min. Cells were then fixed and stained with anti‑APC and anti-TNKS antibodies. Insets show higher magnification fields. Shown here is one illustrative experiment out of two performed. Scale bar, 7 mm.
Lupo et al., Additional File 19: Figure S8
